# AZIN1 level is increased in medulloblastoma and correlates with c-Myc activity and tumor phenotype

**DOI:** 10.1186/s13046-025-03274-1

**Published:** 2025-02-17

**Authors:** Julie Sesen, Tyra Martinez, Sara Busatto, Larysa Poluben, Hassan Nassour, Caroline Stone, Karthik Ashok, Marsha A. Moses, Edward R. Smith, Aram Ghalali

**Affiliations:** 1 Vascular Biology Program, Boston Children’s Hospital, Boston, MA USA; 2 Department of Surgery, Boston Children’s Hospital and Harvard Medical School, Boston, MA USA; 3 Department of Medicine and Cancer Center, Beth Israel Deaconess Medical Center, Harvard Medical School, Boston, MA 02115 USA; 4 Department of Neurosurgery, Boston Children’s Hospital, Harvard Medical School, Boston, MA USA; 5 Vascular Biology Program and Department of Surgery, Boston Children’s Hospital and Harvard Medical School, 300 Longwood Avenue, Boston, MA 02115 USA; 6 Boston Children’s Hospital, Harvard Medical School, Boston, MA USA

**Keywords:** AZIN1, c-Myc, MYC amplification, Medulloblastoma, Extracellular Vesicles

## Abstract

**Background:**

AZIN1 is a cell cycle regulator that is upregulated in a variety of cancers. AZIN1 overexpression can induce a more aggressive tumor phenotype via increased binding and resultant inhibition of antizyme. Antizyme is a protein that normally functions as an anti-tumor regulator that facilitates the deactivation of several growth-promoting proteins including c-Myc. MYC plays a critical role in medulloblastoma pathogenesis. Its amplification serves as a defining characteristic of group 3 medulloblastomas, associated with the most aggressive clinical course, greater frequency of metastases, and shorter survival times.

**Methods:**

Medulloblastoma tissues (68 TMA, and 45 fresh tissues, and 31 controls) were stained (fluorescence and immunohistochemical) for AZIN1. Western blotting and ELISA were used to detect the AZIN1 level. Phenotypically aggressive cellular features were measured by increased invasion, colony formation and proliferation. CRISPR-Cas9-mediated AZIN1 knocked-out cells were orthotopically implanted in the cerebellum of nude mice (*n* = 8/group) with a stereotactic frame. Tumor growth was monitored using the In Vivo Imaging System (IVIS).

**Results:**

Here, we investigated the role of AZIN1 expression in medulloblastoma. We found that overexpression of AZIN1 in medulloblastoma cells induces phenotypically aggressive features. Conducting in vivo studies we found that knocking-out AZIN1 in tumors corresponds with reduced tumor progression and prolonged survival. Clinical specimens are revealing that AZIN1 is highly expressed and directly correlates with MYC amplification status in patients.

**Conclusion:**

These data implicate AZIN1 as a putative regulator of medulloblastoma pathogenesis and suggest that it may have clinical application as both a biomarker and novel therapeutic target.

**Supplementary Information:**

The online version contains supplementary material available at 10.1186/s13046-025-03274-1.

## Introduction

Despite recent progress in characterizing the molecular determinants of tumor growth and progression, current approaches to medulloblastoma (MB) treatment only provide long-term benefits in about half of the affected children [1], as adjuvant therapies such as radiation and chemotherapy remain limited in specificity and efficacy. Currently, commonly used chemotherapeutic agents with relatively nonspecific mechanisms of action have been applied to a small number of disease targets and relatively few new targets have been identified. Consequently, there is a critical, immediate need to identify new druggable targets to treat MB, particularly for the most aggressive group 3 subgroup [2–8], which remains the most refractory to treatment efforts.

One novel potential target relevant to MB is antizyme inhibitor 1 (AZIN1). AZIN1 is a cell cycle regulator that has been found to be upregulated in a variety of cancers. AZIN1 overexpression leads to a more aggressive tumor phenotype by binding to – and inhibiting – its target, antizyme [9]. Normally, antizyme serves as an anti-tumor regulatory agent by facilitating ubiquitin-independent degradation of several growth-promoting proteins including ornithine decarboxylase (ODC), cyclin D1, SMAD1, MSP1, Aurora A kinase and MYC [9]. Basically, AZIN1 is upstream of ODC [9], which is a known transcriptional target of MYC [10], and ODC/polyamine synthesis are dependent on the MYC activity [11], while sufficient access to cellular polyamines is important for MYC activity [12–14] indicating a crosstalk relationship between polyamine synthesis and the MYC oncogene. Therefore, targeting the polyamine regulator AZIN1 might correlate to the MYC activity [11].

Furthermore, AZIN1/antizyme regulation of growth proteins is especially important as it relates to the unique role MYC plays in the most aggressive subgroup of MB, group 3 [8, 15, 16], which is defined by MYC amplification and clinically manifests high rates of metastasis [17], and concomitant poor prognosis [11, 13, 14, 18, 19]. Antizyme antagonizes ODC [20] (the rate-limiting enzyme in polyamine synthesis) both directly and by inducing ODC degradation [9]. This is critical, since ODC activity has been shown to correlate directly with the MYC oncogene family member, MYCN activity in neuroblastoma [21]. In summary, increased AZIN1 and therefore less antizyme leads to more ODC activity and subsequent increases in c-Myc. Thus, a strategy focused on blocking AZIN1 (or increasing antizyme) might represent a novel approach with a sound mechanistic foundation that is uniquely suited for treating the MYC overactivity that defines the subgroup of MB with the poorest prognosis.

In addition to the substantive role antizyme plays in the regulation of c-Myc, antizyme also binds and degrades other cell growth regulators, including cyclin D1 [22, 23] and SMAD1, both of whose expression correlates with worsened prognosis in MB and promotes MB invasiveness [24, 25]. Taken together, these data support the strategy of using an AZIN1 blocker/inhibitor/suppressor as a potential therapeutic approach useful in treating MB.

In this study, we establish that overexpression of AZIN1 in MB cell lines induces the phenotypically aggressive features of increased invasion, colony formation, and proliferation. These in vitro data are corroborated with clinical specimens demonstrating that AZIN1 is highly expressed in the most aggressive subgroup of MB. Within the context of our patient-derived data, we observed that expressions of *AZIN1* and *c-Myc* directly correlates in MB patient samples thereby reinforcing our mechanistic hypothesis in a clinical setting. Mechanistically, we report that *AZIN1* is a transcriptional target for the c-Myc transcription factor which can also explain the high AZIN1 level in MYC amplified MB. Furthermore, we show that AZIN1 exhibits potential utility as a biomarker, with evidence revealing that AZIN1 is highly expressed in the cerebrospinal fluid (CSF) of MB patients relative to age and sex matched controls. In a proof-of-principle study, we found decreased AZIN1 levels in the urine of two patients undergoing surgical removal of the tumor. In combination, these data implicate AZIN1 as a significant factor in the pathogenesis of MB and suggest that AZIN1 may have clinical application as both a biomarker and a novel therapeutic target.

## Material and methods

### Cell culture

D458, D556 and SVG (human fetal glial cell line) cell lines were purchased from ATCC (Manassas, VA). The D283 cells were generously supplied by Dr. R. Jain (from the Massachusetts General Hospital (MGH), Boston, MA). Cells were cultured in DMEM/F12 media (Gibco, cat# 11320), supplemented with 10% Fetal Bovine Serum (FBS) (R&D S11550), 1% penicillin‒streptomycin (Gibco, cat#15140), and 1mL of Normocin (InvivoGen, cat# ant-nr-1).

### Reagents

PolyamineRed was purchased from Diagnocine NJ, USA (cat# FNK-FDV-0020). DAPI was used to stain cell nuclei and is from ThermoFisher Scientific (cat# 62248).

### Extracellular vesicles (EV) isolation and characterization

D556 and D556 KO for AZIN1 cells were cultured for 48 h in complete media supplemented with EV-free FBS (exosome-depleted FBS; System Biosciences, Palo Alto, CA, USA) to produce conditioned media containing secreted EVs. Small extracellular vesicles were isolated from conditioned media using a differential centrifugation protocol previously described by us [26–28]. Briefly, conditioned media was collected and centrifuged at 400 g for 10 min to remove dead cells and large debris, and then at 2,000 g for 20 min and 14,000 g for 45 min at 4 °C (Sorvall Lynx 4000, Thermo Fisher Scientific Inc.) to collect representative samples of apoptotic bodies and larger microvesicles, respectively. The supernatant was subsequently ultracentrifuged at 100,000 g for 90 min at 4 °C (Optima XE-90 Ultracentrifuge, Beckman Coulter Life Sciences). The pellet was suspended in 38 mL of sterile cell-grade PBS and ultracentrifuged again at 100,000 g for 90 min. The final pellet containing washed D556 and D556KO small EVs were suspended in sterile cell-grade PBS (100–250 µL) for characterization and experiments or stored at −80 C. The resulting apoptotic body, large EV, and small EV samples were characterized according to the latest International Society for Extracellular Vesicles guidelines [29]. Sample size and concentration were measured by nanoparticle tracking analysis (NTA, NanoSight NS300, Malvern Instruments, UK). EV formulations were diluted (1:100-1:1000) in 3 mL of PBS and analyzed with a Malvern Panalytical Nanosight NS300 (60-second measurement; 3 capture replicates). The presence of characteristic markers, such as cluster of differentiation (CD) 63, Alix and the absence of GM130, a Golgi marker, as a negative control, was evaluated by Immunoblot (5–20 µg of total proteins/lane as determined by Bradford assay). EV morphology and the association of AZIN1 to the EV structure were evaluated by transmission electron microscopy (TEM). Freshly isolated EVs were adsorbed onto a Formvar/carbon-coated grid, stained with uranyl formate and immunolabeled with two different antibodies against human AZIN1 (1- from Proteintech Group, Inc. Chicago, IL, (cat# 11548-1-AP), and 2- Sigma Aldrich, Saint Louis, MO (cat# WH0051582M1)). The grids were imaged using a JEOL 1200EX TEM and images were taken with an AMT 2k CCD camera. Imaged by TEM and analyzed by NTA, D556- and D556 AZIN1 KO-EVs showed characteristic disc-shaped morphology and heterogeneous size distribution in the nanometer range. D556-EVs and D556 AZIN1 KO-EVs were enriched in typical EV markers, such as Alix and CD63. Both EV samples lacked GM130, a Golgi marker used to detect the possible presence of nanosized intracytoplasmic contaminants.

### Confocal microscopy

Fixed cells (in 4% formaldehyde) were analyzed with an LSM 880 META confocal laser scanning microscope (Zeiss, Oberkochen, Germany) equipped with objectives such as 63x and 40x A-Plan oil immersion. We used ZEN imaging software in multitrack mode to obtain images.

### Plasmid transfection (transient and stable shRNA)

Cells were transfected for 24 h with 2.5 µg plasmid per 60 mm dish using pcDNA3.1 or pcDNA3.1-Clover-AZIN1 using the transfection reagent Lipofectamine^R^ 3000 according to the manufacturer’s protocol (Invitrogen, Carlsbad, CA, USA). The plasmids were constructed as described in [30]. shRNA lentiviral particles for human AZIN1 (cat# sc-77594-V), c-Myc (cat# sc-29226-V) and shRINA Control (cat# sc-108080) were purchased from Santa Cruz Biotechnology Inc. TX, USA. The cells were treated with lentiviral particles, followed by selection with 1 µg/mL puromycin. Knockdown was confirmed by western blot analysis.

### Western blotting

Protein concentrations in lysates, CSF, or urine were measured by Bovine Serum Albumin (BSA) assay and equal amount of proteins were loaded to sodium dodecyl sulfate–polyacrylamide gel electrophoresis (SDS‒PAGE). After the separation step, proteins were transferred onto either a nitrocellulose membrane (Bio-Rad Laboratories, Inc cat# 1620115) or a polyvinylidene difluoride (PVDF) membrane (Bio-Rad Laboratories, Inc cat# 1620177). Membranes were blocked in 5% dry milk (Bio-Rad Laboratories, Inc cat# 1706404) in TBS-T for an hour on a rocker at room temperature. After blocking, membranes were incubated overnight at 4 °C in primary antibody: AZIN1 (Proteintech Group, Inc. Chicago, IL, cat# 11548-1-AP), AZIN1 (Sigma Aldrich, Saint Louis, MO, cat# WH0051582M1), AZIN1 (Abnova, Taipei, Taiwan, cat# H00051582-D01P), CD63 (Abcam, Cambridge, UK, cat# ab68418), Antizyme (Abcam, Cambridge, UK, cat# AB223481), GM130 (GeneTex Inc., CA, USA, cat# GTX130351), c-Myc (R&D Systems, Minneapolis, MN, cat# AF3696), c-Myc (Cell Signaling Technology Inc. Danvers, MA, cat# 5605), ODC (R&D Systems, Minneapolis, MN, cat# 52238), Alix (Cell Signaling Technology Inc. Danvers, MA, cat# 2171S), and/or GAPDH (Cell Signaling Technology Inc. Danvers, MA, cat# 2118). After incubating in primary antibody overnight, the membranes were washed in TBS-T and then incubated in an HRP secondary antibody: Rabbit anti-Goat IgG (Thermo Fisher Scientific, Waltham, MA, cat# A27014), Goat anti-Rabbit IgG (Thermo Fisher Scientific, Waltham, MA, cat# 31460), and/or Goat anti-Mouse IgG (Thermo Fisher Scientific, Waltham, MA, cat# 31430). Membranes were incubated in secondary antibody for an hour at room temperature on a rocker. Luminescence was visualized with Enhanced Chemiluminescence Substrate (ECL) (VWR International, LLC, cat# 10061-494). The results of western blotting were analyzed with NIH ImageJ 1.62 software. The extracellular protein precipitation was performed by using acetone according to the protocol for acetone precipitation of protein (Pierce Biotechnology, Thermo Fisher Scientific, Waltham, MA).

### Proliferation assay

Cells were seeded in 96-well plates at 5,000 cells per well in 100 µl medium. After overnight incubation at 37 C˚, 5% CO_2_, each well of the 0 h plate was incubated with 10 µL Cell Counting Kit-8 assay (Dojindo Molecular Technologies, Inc., Rockville MD) for 2 h away from light before measuring the absorbance at 450 nm by a microplate reader. The other plates were incubated for the indicated hours (24, 48 and/or 72 h), followed by adding the Cell Counting Kit-8 solution for 2 h away from light and measuring the absorbance at 450 nm by a microplate reader.

#### Proliferation assay after EV treatment

D556 cells were seeded in a clear 96-well plate treated daily with a fixed dose of EVs (10^3^ EVs/cell) or an equal volume of PBS and kept in culture for a total of 24, 48, 72, and 96 h. At the end of each time point, cell viability was measured by 3-(4,5-dimethylthiazol-2-yl)−5-(3-carboxymethoxyphenyl)−2-(4-sulfophenyl)−2 H-tetrazolium (MTS) assay. MTS reagent (20% of cell media volume) was added to the cells and incubated at 37 °C for 1 h before measuring absorbance at 490 nm using a microplate reader.

### Invasion assay

Invasion assays were performed using Corning Transwell Permeable Polycarbonate Membrane Inserts with an 8.0-µm pore size and coated with BD Matrigel Basement Membrane Matrix (BD Biosciences; cat # 354483) as previously described by us [31]. D556 MB-cells were treated once with a fixed dose of AZIN1-EVs, AZIN1KO-EVs (10^3^ EVs/cell), or a correspondent volume of PBS. After 24 h, cells that had migrated through the filters after 20 h at 37 °C were stained with the Diff-Quick Cell Staining Kit (Dade Behring Inc.), and four fields were counted by phase microscopy.

### Soft agar colony formation assays

A total of 5 × 10^3^ cells in 0.4% Bacto agar were seeded on top of a solidified layer of 0.6% Bacto agar in 6-well plates. Colonies were counted after 19-21 days, and the data was expressed as the mean ± s.e.m. of three independent wells from the same experiment.

### Patient population

CSF, tissue and urine were collected as previously published [32–34] and in accordance with protocols approved by the Boston Children’s Hospital IRB (IRB#10–417); informed consent was obtained.

Pediatric MB patients (age 18 years and younger; *n* = 45) presented with tumors on magnetic resonance imaging studies and a tumor diagnosis was confirmed as part of standard clinical practice with neuropathology. The pediatric patients didn’t present with a prior history of untreated tumors, vascular malformations or recent surgery within 3 months of specimen collection. Tissue specimens (*n* = 45) were obtained from the Division of Neuropathology at BCH. After collection the tumor tissue was embedded in paraffin. Subsequently, the tissue was cut into 5-µm-thick sections and mounted on microscope slides.

Control CSF (*n* = 8) from patients (age 18 years and younger) who had fatty filum and normal CNS imaging studies and pediatric patient CSF (*n* = 11) was collected before surgery. Pediatric patient urine (*n* = 2) was collected before surgery. A second urine collection from the same pediatric patient was performed 8 weeks after surgery for patient 1 (11 weeks for patient 2) and served as a control urine sample.

### Tumor collection and dissociation

Samples were collected after patient consent and in accordance with Dana–Farber/Harvard Cancer Center protocol #10-417. Information about this protocol/consent document are available on the ODQ Neuro-Oncology database.

The dissection and methodology of tissue samples extracted during neurological procedures is determined by the performing physicians. All tissue collection is processed by the pathology department and the amount of excess tissue not needed for clinical care purposes is decided by them. CSF may be collected during surgery only if accessible and doesn’t involve additional invasive interventions; these guidelines are determined by the performing physician at the time of surgery. Urine samples may be collected prior to, during, or after routine clinical visits or medical procedures. Urine collections are provided by the patient and do not involve additional invasive interventions. However, the collection of urine after surgery was according to the IRB protocol that is in Title: Boston Research, Academics, & Innovation in Neurosurgery (B.R.A.I.N.) Biorepository and Registry, at Boston Children’s Hospital (IRB-P00044060).

### Patients and tissue microarray (TMA)

We purchased and stained (for AZIN1) 25 cases of brain tissues and used them in the immunohistochemistry experiments (below) from TissueArray.Com LLC (US Biomax, Inc.) (cat# BNC17011d). We also stained 38 cases of astrocytoma, 22 MB, 14 glioblastoma, 6 oligodendroglioma, 1 ependymoma, 3 cancer adjacent brain tissue and 3 normal brain tissues obtained from TissueArray.Com LLC (US Biomax, Inc.) (cat# BC17012c and BS17015b). We established a collaboration with the Children’s Oncology Group (COG) (approved protocol number: ACNS20B1-Q), and they provided 47 additional cases of MB which were also stained.

### Immunohistochemistry

TMAs or paraffin embedded slides were deparaffinized with xylene, rehydrated, and subjected to brief proteolytic digestion and peroxidase blocking. All fresh tissues were processed by our core histopathology laboratory as part of clinical care and neuropathology review to confirm diagnosis. Slides were incubated overnight at 4 °C with a 1:400 dilution of a polyclonal AZIN1 antibody (11548-1-AP; Proteintech Group, Inc. Chicago, IL). We used AZIN1 overexpression (mammalian expression vector) to validate the AZIN1 antibodies. Tissue sections that were formalin-fixed, paraffin-embedded were mounted on 25 × 75 × 1.0 mm microscope slides. Using the Closed Loop Assay Development (CLAD) manufacturer instructions (Ventana Medical Systems), the indicated antibodies were optimized by using the OmniMap DAB Anti-Rabbit (HRP) Detection Kit (Ventana Medical Systems). Standard quality control procedures were undertaken to optimize antigen retrieval, primary antibody dilution, secondary antibody detection and other factors for both “signal and noise.” No primary antibody treatment was used as negative controls.

### Immunofluorescence and histological analysis

Paraffin-embedded tumor tissues were cut into 5-µm sections, were stained for histopathological analysis using Hematoxylin and Eosin (H&E) staining and imaged using an inverted light microscope at 20x and 40x objectives (Zeiss Axio Observer Z1; Carl Zeiss Microscopy, Thornwood, NY). For immunofluorescence, tissues were dissected and maintained in 4% paraformaldehyde (PFA, for 24 h) and placed in 30% sucrose (for 72 h). Tissue was embedded in optimum cutting temperature reagent (Tissue-Plus O.C.T.; Fisher Healthcare, ThermoFisher Scientific) were cut into 5-µm sections. To deparaffinize the paraffin-sectioned slides, we used xylene and rehydrated in stepwise decrease of ethanol concentration followed by water. Before antigen retrieval, slides were fixed in 10% neutral buffered formalin for 30 min. Antigen retrieval was performed in antigen retrieval buffer (10mM Tris-HCl, 1mM EDTA with 10% glycerol [pH 9]) at 110 °C for 17 min (∼4–5ψ). Slides were left at room temperature to cool down and were then washed with PBS (once for 15 min). Tissue sections were blocked with 2.5% goat serum (Vector Laboratories, S-1012) for 90 min followed by incubation with primary antibody overnight: AZIN1 (1:200; cat# 11548-1-AP; Proteintech Group, Inc. Chicago, IL), c-Myc (1:200; cat# AF3696; R&D Systems, Minneapolis, MN). After washing, the slides were incubated with HRP-conjugated secondary Antibodies 1:1000 (Alexa Flour 488 donkey anti-goat (cat# A11055), Alexa Fluor 594 Goat anti-Rabbit (cat# A11012 and A11037) from Invitrogen by ThermoFisher Scientific, for 60 min on a shaker. Counterstaining was performed using DAPI, and then cover slips were added to the slide were added using ProLong Gold mount (no. P36931; Life Technologies). Images were taken and processed on a confocal microscope using 20x objective (Zeiss LSM 880 laser scanning microscope with Airyscan; Carl Zeiss Microscopy).

### The enzyme-linked immunosorbent assay (ELISA)

AZIN1 (cat# MBS9314800) ELISA kit was purchased from MyBioSource Inc. CA, USA. ELISA was performed according to manufacturer instructions. The optical density (OD) was measured with FilterMax F3 spectrophotometer (Molecular Devices) at a wavelength of 450 nm, and the concentration of AZIN1 in samples was calculated by using the standard curve as a template to compare the samples OD against. Results of AZIN1 levels in the samples were compared to levels from control samples (as indicated in the figure legends) and normalized to total protein concentrations. Protein concentrations are given in nanograms per microgram (ng/mg) and determined by dividing the concentration of the target protein in the sample (ng/mL) by the concentration of total protein in the sample (µg/mL). The detection range of the assay goes from 0.625 ng/ ml to 20 ng/ml and the sensitivity is 0.1 ng/ml.

### Chromatin immunoprecipitation (ChIP) assay

ChIP-PCR was performed as previously described [35]. The cells were cultured and plated to ensure they reached a healthy confluency of 90% on 10 cm plates before crosslinking. Briefly, cells were crosslinked with 1% formaldehyde (single fixation) or 2 mM fresh disuccinimidyl glutarate and 1% formaldehyde (single fixation) and then chromatin sonicated to 200–600 bp. ChIP was carried out using antibody against c-Myc (D84C12) (Cell Signaling, cat # 5605S). ChIP DNA was purified using the SimpleChIP^®^ Plus Enzymatic Chromatin IP Kit (Cell signaling, cat #9005). The ChIP-DNA was analyzed by qPCR using SimpleChIP^®^ Universal qPCR Master Mix (Cell Signaling, cat # 88989) according to the manufacturer’s instructions. Primers that are targeting regions of interest are listed in Table 1.

**Table 1:** Primers used for the ChIP assay

Targeting Site	Direction	Primer Codon
Promoter pair 1	Forward	GGATTCGGACTCGTCACACT
Promoter pair 1	Reverse	CTTTCAGGCTCTGATCGCGG
Promoter pair 2	Forward	AGATAACGGCCCAAAGAGTCG
Promoter pair 2	Reverse	CGCTTTTACCCCCAAGTCGT
Promoter pair 3	Forward	TGGTGACATCACAGCTGGC
Promoter pair 3	Reverse	GCATGACCCGGTCGC
Reported regulatory region (MYC binding site) pair 1	Forward	ATGCAGAGCCCTGTTGAGTC
Reported regulatory region (MYC binding site) pair 1	Reverse	CCCAGTGTGCTTCTCTGTGT
Reported regulatory region (MYC binding site) pair 2	Forward	GCCAATCTTTGCTGCTTCCC
Reported regulatory region (MYC binding site) pair 2	Reverse	AGAAGCCATGTGGTCTGTGG
Gene body pair1	Forward	CCAAAAGTTATAAGCCCAACAGGC
Gene body pair1	Reverse	GGTGAGGCAGTTCATTGGGC
Gene body pair2	Forward	TCACGTGAGATAACGGCCC
Gene body pair 2	Reverse	CCCTCTTTAAAAATTCCGCCG

As determined by qPCR, following primers were efficient and were used for further studies; promoter pair 2, reported MYC binding site pair 1 and gene body pair 2

### CRISPR AZIN1 knock out

The D556 AZIN1 KO cells were generated by Alstem Inc. (AlstemBio. CA, USA). In short, we targeted exon 3 (the first coding exon) that is shared among all longer transcripts. We designed two gRNA pairs within this exon to delete a portion of the coding sequence, by introducing frame shift mutations, (Supplementary Fig. 3A and B). sgRNAs were cloned into an expression vector, and transfected into D556-Luc-GFP cell line using Lipofectamine 3000. Used gRNAs were: sgRNA 1: TGGAATACGGCTGAGATGAA, sgRNA 2: TCCTTCATCCAACAGGCCAA, sgRNA 3: TTGAGAAATGGCACTTACCA. After transfection, the knockout efficiency of gRNA pairs was determined by PCR using genomic DNA from the transfected pooled cells. Primers that were used: AZIN1 F: AGCACACTTTCATTGACTCGT, AZIN1 R: TGTGGGTGCCAGATGCTAAA After efficient cutting was confirmed, single cells from the transfected pooled cells were seeded to expand into homogenous colonies (Supplementary Fig. 3A-D). Single clones were collected for knockout mutations screening by PCR followed by DNA sequencing. After screening ~ 50 single-cell clones, we have obtained 4 homozygous AZIN1 knockout clones. Clones; E4, E5, F8 and C6 showed frame shifts Supplementary Fig. 3C, only clone E5 still express the AZIN1 protein Fig. 4A.

### Single-cell RNA sequencing analysis

#### Data retrieval and processing

The expression count matrix encompassing 36 medulloblastomas from all molecular subgroups (WNT, SHH, Group 3, and Group 4 tumors) were retrieved from GEO accession GSE119926 [36]. Samples from 11 PDX model and two samples with fewer than 100 cells were excluded from downstream analysis. This resulted in an expression matrix of 23 samples, which was imported into R (version 4.2.3) and used to create a Seurat object (Seurat v4.3.0) [37, 38].

#### Data integration and dimensionality reduction

All cells from the four molecular subgroups were merged and integrated for analysis using the Harmony integration method [39]. Integration was performed to correct for batch effects and achieve a unified dataset for clustering and annotation. Principal component analysis (PCA) was conducted over the 2000 most highly variable genes to reduce data dimensionality, with the top 20 principal components selected for downstream analysis. Cell clustering was performed on integrated data using a shared nearest-neighbor (SNN) graph-based method using the FindNeighbors function included in Seurat, followed by modularity optimization using the Louvain algorithm (FindClusters) with a resolution parameter of 1.

#### Identification of MYC-positive and MYC-negative cells

To identify the cells expressing MYC, log normalized expression value of MYC gene were extracted from the integrated dataset. Cells were classified as MYC-positive if their log-normalized MYC expression values were greater than zero and MYC-negative otherwise.

#### Analysis of AZIN1 expression in MYC positive

The expression level of AZIN1 was compared across the molecular subgroups for the MYC-positive. Pairwise statistical comparisons between groups were performed using the Student's t-test. Additionally, a one-way ANOVA-Kruskal-Wallis test was applied to evaluate significant differences in AZIN1 expression across all groups. Following significant ANOVA results, post-hoc tests were conducted for pairwise comparison between subgroups to identify differences.

### Medulloblastoma xenografts (intracranial injection)

All mouse studies were approved and performed in accordance with the policies and regulations of the Animal Care and Use Committee of Boston Children’s Hospital. Intracranial MB xenografts were established by injecting WT or AZIN1 KO GFP-Luciferse-D556 cells into the cerebellums of Athymic nude mice nu/nu (The Jackson Laboratory - Stock No: 002019 inbred)– (immunosuppressed line of mice lacking T cells). Cerebellar coordinates were − 2 mm from lambda, + 1 mm laterally, and 3.0 mm deep. Tumor growth was evaluated on days 0, 7, 9, 14 and before sacking, by bioluminescence imaging using an in vivo spectral imaging system (IVIS Lumina II, Xenogen). They were sacrificed if they showed signs of pain, including vocalization when handled, lethargy, or hunched posture. Additional criteria that could result were focal neurologic symptoms such as weakness, ataxia seizures or signs of excessive discomfort. Additionally, if an animal appeared in poor health and had weight changes such as: 10% weight loss/day or a > 20% weight loss compared to baseline, then the animal was sacrificed.

### Generation of mouse Group3 medulloblastoma

Mice were generated as described in [4]. In short, the cerebellar granule neural precursor cells were purified from P7 cerebella of [Ink4c-/-; p53-/-] mice. They were infected in vitro with a retrovirus expressing Myc-RFP and then implanted into the brains of recipient nude mice. Cells from mice that developed brain tumors were passaged into nude mice to amplify those lines in vivo.

### Analyses of public available transcriptomics data

We used the R2 genomics platform (http://r2.amc.nl) to identify suitable transcriptomics datasets with molecular and histological subgroups available for MB. We selected the following datasets [8, 40–43]: Pfister (167 samples, fpkm normalized, mb500rs1 chip), Korshunov (420 samples) FFPE Group 3/4 - RPKM – mbffpe), Pfister − 223 - MAS5.0 - u133p2, and Cavalli (763 samples, rma_sketch normalized, hugene11t chip). For each of them, we download log2 transformed and normalized data through the data grabber function. In Cavalli for each subgroup (SHH, Group4, Group3 and WNT) expression levels.

### Statistical analysis

Statistical analysis was conducted using the Student’s t-test. For data that did not follow a normal distribution, nonparametric Mann-Whitney U tests were used. For correlation studies, simple linear regression analyses were used. The data were presented as mean ± SD. Experiments were performed at least three times. For a result to be deem as statistically significant, a *p*-value needed to be: *p* ≤ 0.05. Statistical analysis for single-cell RNA sequencing analysis is given in the materials and methods description of that method.

## Results

### AZIN1 is present in clinical patient tumor samples and its levels correlate with MYC expression

**Fig. 1:**
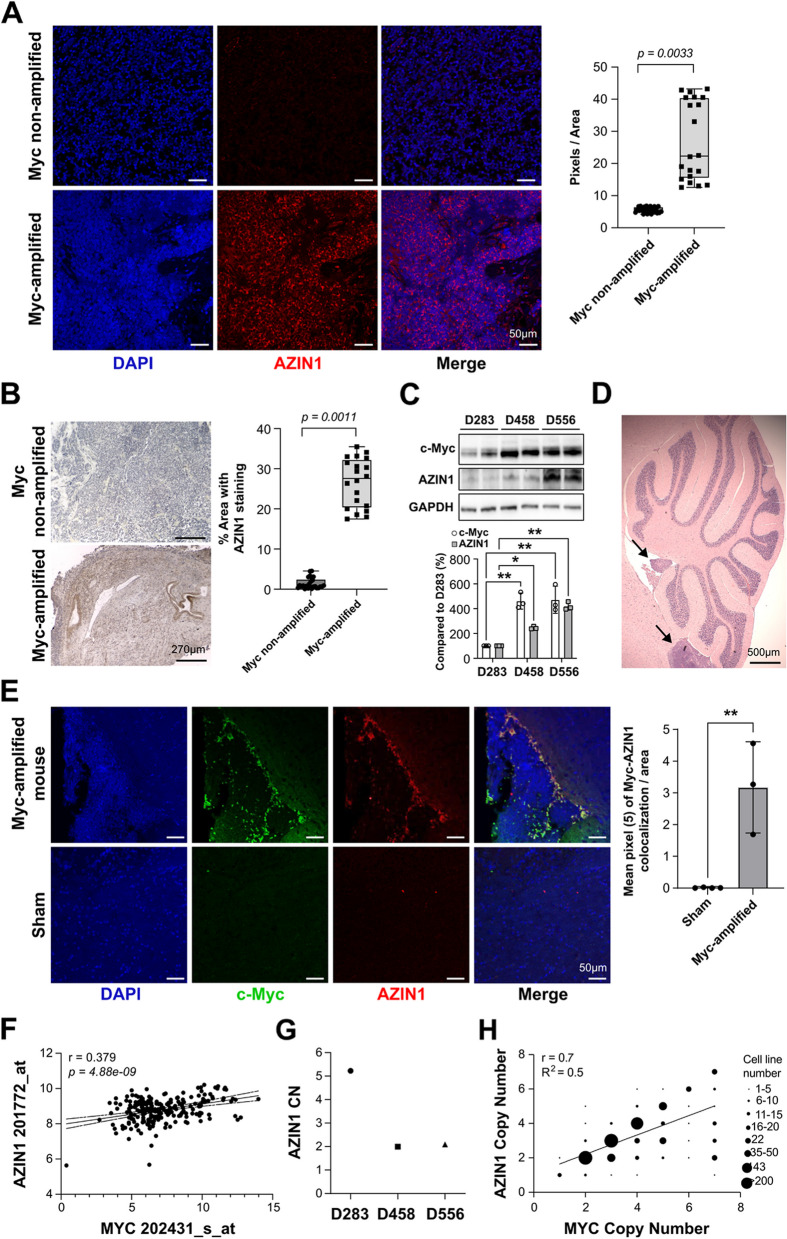

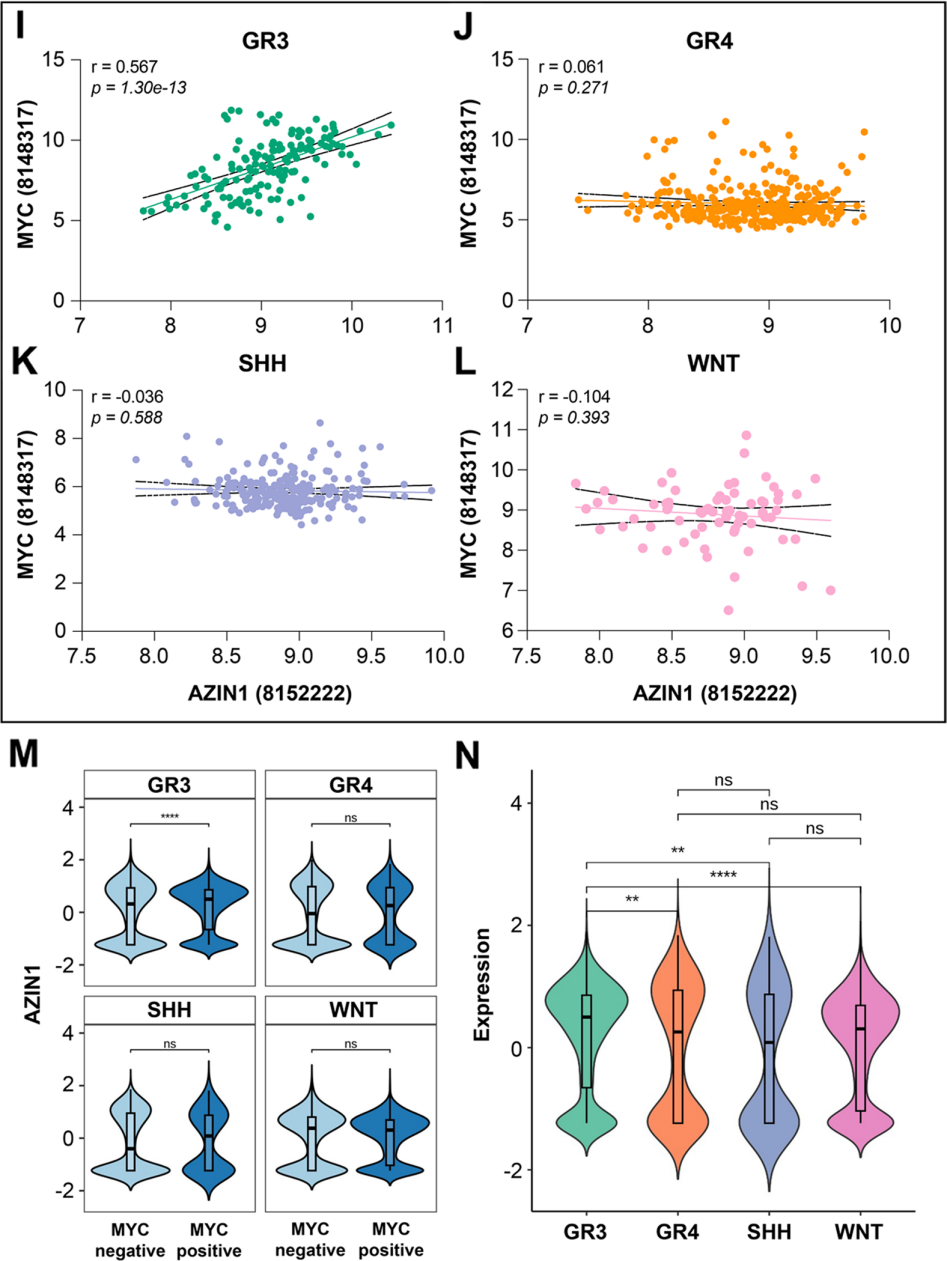
AZIN1 expression is increased in c-Myc amplified MB patients. MB fresh tissues from MYC amplified patients (*n* = 25) and MYC non amplified patients (*n* = 20) were stained for AZIN1; in **A** the immunofluorescence signal was detected by confocal microscopy and in **B** Immunohistochemical staining was performed, and images were taken on the ECHO Rebel-18 Microscope at 4X objective. **C** The levels of c-Myc and AZIN1 were measured by Western Blotting (WB) in MB cell lines for indicated antibodies and GAPDH was used as loading control. Bars represent the mean (±S.D.) from three independent experiments. Significant difference to corresponding proteins in D283 * (*p* < 0.05), **(*p* < 0.01). **D** H&E staining from a representative mouse brain resembling MYC amplified MB mouse model (*n*=3). **E** Immunofluorescence of slides from brain of MYC amplified MB mouse model (*n*=3) and sham mice brains (*n* = 4) were used as controls. Sham mice underwent intracranial injection, but instead of cells, sterile PBS were injected. Bars represent the mean (±S.D.) from three independent experiments. ** Significant difference to sham (*p* < 0.01). **F** Using publicly available dataset of 223 MB patients [43] and analyzing the correlation between *AZIN1* and *MYC* mRNA expressions (*p*- value 4.88 e-09). **G** The *AZIN1* copy numbers were measured by Copy Number Variation assay in non MYC amplified D283 and MYC amplified D458 and D556 cells. **H** Using publicly available Cancer Cell Line Encyclopedia (CCLE) database, the correlation between *MYC* and *AZIN1* copy numbers in 996 cell lines were analyzed (R = 0.7, *p*-value = 1.90207e^-150^). (**I**-**L**) Data from a publicly available dataset [40] were analyzed for correlation between *AZIN1* and *MYC* mRNA expressions in; group 3 (*p*=1.30e-13) **I**, group 4 (*p*=0.271) **J**, SHH (*p*=0.588) **K** and WNT (*p*=0.393) **L** MB subgroups. **M** Single-Cell RNA Sequencing data [36] from group 3, group 4, SHH and WNT MB subgroups were sorted based on MYC expression status individually and a comparison was performed for each subgroup (Supplementary Table 1A and B). AZIN1 expression was compared between MYC positive cells verses MYC negative cells, among each individual MB subgroup. **N** AZIN1expression in MYC positive cells were compared between MB subgroups using Single-Cell RNA Sequencing data [36] (Supplementary Table C)

We have previously reported that increased AZIN1 expression predicts aggressiveness and poor outcome in prostate cancer [31]. We conducted AZIN1 immunohistochemistry using tissue microarrays composed of 69 cases of MB, 38 cases of astrocytoma, 14 cases of glioblastoma, 6 cases of oligodendroglioma, 1 case of ependymoma, 31 controls (consists of 3 cases of cancer adjacent brain tissue and 28 normal brain tissues). Our initial analysis of these childhood brain cancers revealed variable AZIN1 expression in MB with some patient samples exhibiting high AZIN1 expression while others having no detectable expression. This heterogeneity in AZIN1 levels prompted us to investigate whether AZIN1 expression is specific to subgroup and/or grade of MB. Consequently, we sorted the MB patient samples according to their known MYC amplification status as is done as part of routine clinical tumor subtyping and then performed immunohistochemistry and immunofluorescence on 45 fresh MB tissues. Importantly, we observed a pattern whereby patient tissues with MYC amplification had significantly higher AZIN1 expression when compared to patients without MYC amplification (Fig. 1A and B).

### AZIN1 levels correlate with MB aggressiveness in vitro and in vivo

We further investigated this finding linking AZIN1 and c-Myc levels in vitro using the MB cell lines, D283, D458, and D556. It has been reported that D283 cells do not exhibit MYC amplification [44] while both D458 and D556 do [45]. As shown in Fig. 1C, the AZIN1 data from our experiments corroborate these findings, with concordant higher AZIN1 expression in MYC amplified cell lines. Prior reports indicate that AZIN1 levels directly correlate with increased cellular aggressiveness [31] and our experiments further support this premise, with the lowest AZIN1 expression found in the least aggressive D283 cells, and the highest AZIN1 expression levels observed in the most aggressive D556 cells (the doubling time of D283 were more than twice of D458 and/or D556, measured by proliferation rates).

We next conducted in vivo studies using an established murine model that closely mimics human MYC amplified MB tumors [4]. In this MYC amplified MB mouse model, granule neural precursor cells were purified from the cerebella of [Ink4c-/-; p53-/-] mice. These cells were later infected in vitro with a retrovirus expressing Myc-RFP and then implanted into the cortex of nude mice [4] which developed tumors resembling MYC amplified MB (Fig. 1D). Analyzing the AZIN1 expression in these mice and their tumors, we found that AZIN1 colocalized with c-Myc (Fig. 1E) implicating a possible role for AZIN1 in MB development.

### AZIN1 is highly expressed in medulloblastoma samples and correlate with MYC amplification status in medulloblastoma samples

Our in vivo data, coupled with our in vitro and patient specimen results, led us to ask whether a correlation between MYC and AZIN1 exist in a larger cohort of human MB samples. Using the public dataset with hundreds of verified clinical specimens [43], we found a significant correlation between *AZIN1* and *MYC* mRNA levels in MB tissue analyzed from 223 patients (*R = 0.379, p = 4.88e-09*) (Fig. 1F). Grounded in this correlation, we also analyzed *antizyme (OAZ1)* and *ODC* mRNA levels derived from these same clinical specimens, as these molecules are downstream of AZIN1. We found that their levels also significantly correlated with *MYC* mRNA levels (Supplementary Fig. 1A and B). Expanding on this finding by using other publicly available datasets, we used three additional datasets and confirmed that *AZIN1* and *MYC* mRNA are significantly correlated in MB patients (Supplementary Fig. 1C-E). This correlation was also present in mRNA of 1037 cell lines from the Cancer Cell Line Encyclopedia (CCLE) dataset (Supplementary Fig. 1F).

We next performed a copy number variation analysis and looked for *AZIN1* amplification in the MB cell lines. We found that there are 5 copy numbers of the *AZIN1* gene in the MYC non amplified D258 cell line, while the MYC amplified D458 and D556 cell lines do not have *AZIN1* amplification (Fig. 1G). To establish a clearer understanding about the correlation between amplification status of MYC and AZIN1, we expanded the scope of our study and investigated *MYC* and *AZIN1* gene copy numbers within the CCLE database. The *MYC* and *AZIN1* copy numbers in 996 cell lines were analyzed, and we found that *MYC* and *AZIN1* copy numbers are significantly correlated (*R* = 0.7, *p*-value = 1.90207e-150) (Fig. 1H). Considering only cell lines with MYC amplification (*n* = 638), we found a significant correlation between *MYC* and *AZIN1* status (*R* = 0.49, *p*-value = 4.861007e-40) (Supplementary Fig. 1G). This finding is in line with the correlation between *AZIN1* and *MYC* amplifications in cell lines that harbor only AZIN1-amplification (*n* = 559) (*R* = 0.68, *p*-value = 9.094104e-78) (Supplementary Fig. 1H). Together, these data indicates that *MYC* and *AZIN1* amplification are correlated and that AZIN1 overexpression is present in aggressive human MB samples. Looking at MB subgroups for *AZIN1* and *MYC* correlation, we found *AZIN1* to be significantly correlated with the *MYC* mRNA in the aggressive group 3 MB (*R = 0.567, p* = 1.30e-13) (Fig. 1I), and this was not seen in any of group 4 (*R = 0.061, p* = 0.271), SHH (*R = -0.036, p* = 0.588) or WNT (*R = -0.104, p* = 0.393) subgroups (Fig. 1J-L). Analyzing Single-Cell RNA Sequencing of publicly available datasets [36], we looked at all four MB subgroups and sorted them based on their MYC expressions. Comparing *AZIN1* expression in MYC positive verses MYC negative cells in each MB subgroups individually, we found that only in group 3 (*p* = 0.000023), *AZIN1* expression is significantly higher in MYC positive cells, and this was not seen in the other MB subgroups (group 4 (*p* = 0.37), SHH (*p* = 0.8) and WNT (*p* = 0.4)). (Fig. 1M, Supplementary Table 1 A and B)). Moreover, looking at the overall *AZIN1* expression in MYC positive cells, but this time performing a comparison between the MB subgroups, we found AZIN1 to be significantly higher in group 3 MB compared to the group 4, SHH or WNT (Fig. 1N, Supplementary Table 1 C). Together, these findings indicates that the high AZIN1 expression might be selective to the aggressive group 3 MB.

### Overexpression of AZIN1 results in an aggressive MB cellular phenotype

**Fig. 2:**
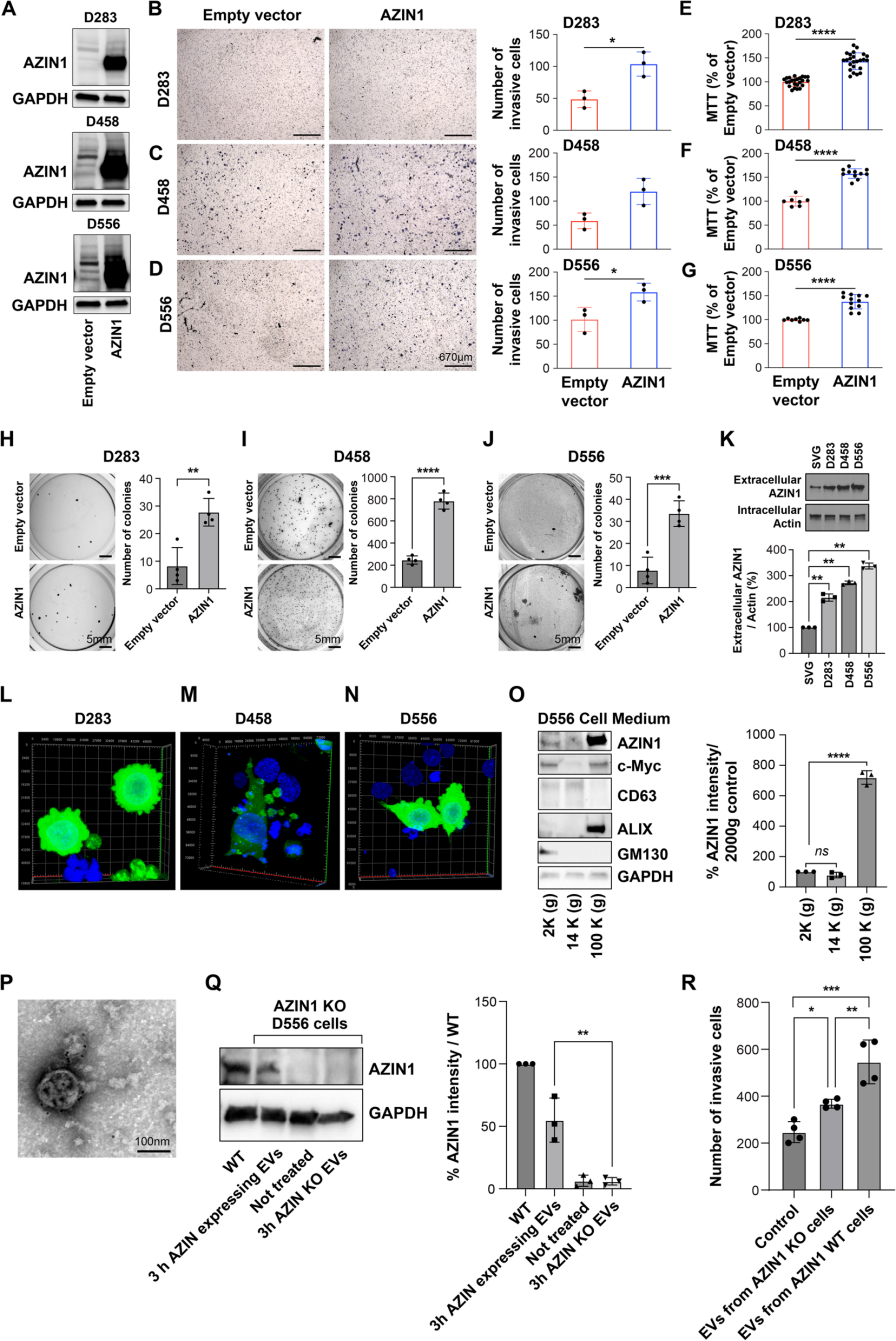
AZIN1 overexpression or transfer by EVs causes a significantly increased MB cell invasion and proliferation. **A** D283, D458 and D556 cells (as indicated in the figure) were transfected with plasmids expressing pCDNA3.1 (Empty) and pCDNA3.1 AZIN1, and the transfection efficiency was measured by WB, using AZIN1 antibody. GAPDH was used as a loading control. The effect of overexpressing AZIN1 plasmid on the behavior of D283, D458 and D556 cells with regard to **B**-**D** invasion into extracellular matrix, **E**-**G** cell proliferation, or **H**-**J** colony formation in soft agar. **A**-**J** MB cells (as indicated in the figure) were transfected with plasmids expressing pCDNA3.1 (Empty) and pCDNA3.1 AZIN1. Proliferation was measured by MTT assay. Invasion was through Matrigel supported by transwells (8 μm pores) followed by methanol fixation and toluene blue staining. Soft agar colony formation assays were performed for 19-21 days, fixed, and crystal violet stained. **K** Normal human fetal glial SVG, D283, D458 and D556 cells were cultured for 24h and serum starved for additional 24h, extracellular AZIN1 in the cell medium was detected by WB and Actin was used as a loading control. **L**-**N** pCDNA3.1-Clover-AZIN1 plasmid was transfected in D283 **L**, D458 **M** and D556 **N** cells and analyzed by confocal microscopy, 3D images are shown. **O** WB analysis confirming that AZIN1 is secreted by MB cells via small EVs (AZIN1-EVs). Apoptotic bodies, large EVs, and small EVs were isolated and analyzed by WB for indicated proteins. **P** TEM images of AZIN1-EVs isolated from D556 cells. **Q** AZIN1-EVs transfer AZIN1 to the AZIN1KO D556 cells as confirmed by WB analysis. **R **AZIN1KO D556 cells treated with AZIN1-EVs exhibit an increase invasiveness in vitro compared to small EVs derived from D556 cells KO for AZIN1 (AZIN1KO-EVs). In **B**-**R**, bars represent the mean (±S.D.) from three independent experiments. * Significant difference to indicated controls (*P* < 0.05), ** (*P*< 0.01), *** (*P*< 0.001), **** (*P*< 0.0001)

To determine whether AZIN1 mRNA results in protein gain of function, we transiently overexpressed AZIN1 in MB cells (Fig. 2A). Increased tumorigenic potential of D283, D458, and D556 MB cell lines was determined by measuring cell proliferation, invasion, and anchorage-independent colony growth. We found that AZIN1 overexpression results in significantly higher cell invasiveness (Fig. 2B-D), proliferation (Fig. 2E-G), and colony formation (Fig. 2H-J) in MB cell lines.

Another AZIN isoform, AZIN2, has been shown to be a secreted protein [46, 47], whereas AZIN1 has not. To investigate whether AZIN1 is released into the extracellular environment, cell culture media from MB cell lines were examined and compared with a normal, but immortalized, human fetal glial cell line, SVG. We found AZIN1 to be present in cell media and at significantly higher concentrations in media of MB cells compared to that of SVG cells (Fig. 2K and Supplementary Fig. 2). Furthermore, the expression of extracellular AZIN1 increases directly with the aggressiveness of the MB cells, in which the D556 cells exhibit the highest AZIN1 secretion. MB cell lines transfected with a Clover (Green Fluorescent Protein (GFP))-tagged AZIN1 plasmid were characterized by pronounced membrane blebbing events which might suggest an increased plasma membrane shedding and subsequent extracellular vesicles release (Fig. 2L-N and supplementary video A-C).

### AZIN1 is secreted in extracellular vesicles (EV)

High levels of AZIN1 in the extracellular environment and the pronounced plasma membrane blebbing of AZIN1 overexpressing cells led us to ask whether AZIN1 is released from MB cells via cell-secreted EVs. We isolated and characterized apoptotic bodies, large EVs, and small EVs from D556 cells to characterize their protein expression. Apoptotic bodies and large D556-EVs were positive for CD63 whereas small D556-EVs were positive for Alix, both known EV markers [26, 27, 29]. Importantly, we were able to detect, for the first time, AZIN1 in small D556-EVs (Fig. 2O). We also detected c-Myc in apoptotic bodies and small EVs. TEM immunolabeling further confirmed that AZIN1 was located on the membrane of small EVs (AZIN1-EVs) (Fig. 2P).

Subsequent to the discovery of AZIN1 in D556 small EVs, we sought to explore the potential implication(s) and role(s) that AZIN1-EVs may have in MB pathogenesis. AZIN1 was successfully knocked out (KO) in D556 cells (Supplementary Fig. 3A-D and Fig. 4A) and AZIN1-EVs from WT D556 and EVs from AZIN1 knockout (KO) D556 cells (AZIN1KO-EVs) were used to treat AZIN1 KO D556 cells in order to determine whether AZIN1-EVs can deliver functional AZIN1 protein. Our data confirms that AZIN1-EVs are taken up by target D556 AZIN1-KO cells and that functional AZIN1 protein is transferred by circulating EVs (Fig. 2Q). Next, we examined the effect of AZIN1-EVs on WT D556 cell proliferation and invasion. We found that AZIN1-EVs significantly enhance the invasive capacity of D556 cells when compared to cells treated with AZIN1KO-EVs (Fig. 2R) while cell proliferation was not affected (Supplementary Fig. 3E). These findings demonstrate that AZIN1 can be transferred via EVs to neighboring cells inducing an increased cancer cell invasion characteristic of highly aggressive MB cells. These data also suggest that AZIN1-EVs may be implicated in MB invasion and dissemination.

### AZIN1 KO decreases tumor growth and prolongs survival in vivo

**Fig. 3:**
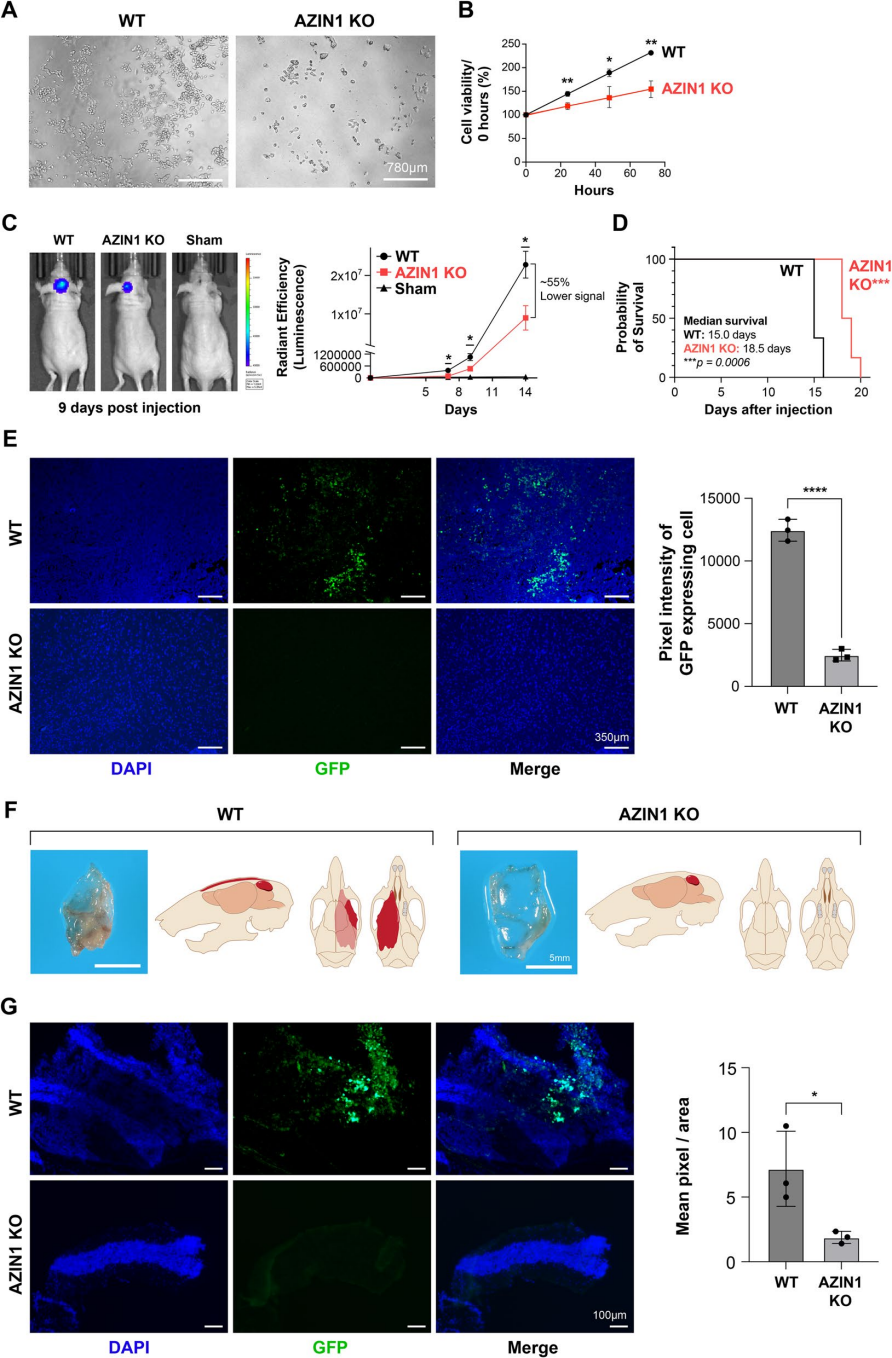
AZIN1 CRISPR KO decreases tumor growth and prolongs survival in vivo. Using CRISPR/Cas9 gene editing and specific sgRNAs, AZIN1 protein expression was successfully knocked out in D556 cells; **A** Cell growths were monitored by microscopy, images taken by the ECHO Rebel-18 Microscope at 4X objective after 72 hours. **B** Cell proliferation was measured by MTT assay. **C** GFP-Luciferase expressing D556 WT or AZIN1 KO cells were injected intracranially into nude mice and tumor growth were measured by AVIS Spectrum in vivo imaging system. In **D** the survival of the mice is presented in a Kaplan-Meier curve. **C** and **D** WT and KO (*n*=8/group; 4 females and 4 males), sham (*n*=3, male mice) at 14 days post injection, 7/8 mice in both WT and KO groups showed visible tumors and were included in the study. **E** Images from mice brain sections, 14 days post-injection, stained with DAPI and excited by the 488 nm laser to detect GFP expressing cells, without the use of antibodies. **F** Schematic images of inner frontal part of mice skull after being injected intracranially with WT or AZIN1 KO D556 for 15 days. **G** 5µM sections from the inner frontal part were stained with DAPI and excited by the 488 nm laser to detect GFP expressing cells, without the use of antibodies. In **B**-**G**, bars represent the mean (±S.D.) from three independent experiments. * Significant difference to indicated controls (*P* < 0.05), ** (*P*< 0.01), *** (*P*< 0.001), **** (*P*< 0.0001)

CRISPR-Cas9-mediated AZIN1 KO D556 cells were cultured and compared to D556 wildtype (WT) cells. Observing the cells by microscopy, there was less cell growth in the AZIN1 KO line (Fig. 3A). Measuring cellular proliferation by using (MTT) assay, we found that the AZIN1 KO cells had a significantly reduced proliferation rate relative to controls (Fig. 3B). AZIN1 WT and KO cells were orthotopically implanted in the cerebellum of 4-6 week old nude mice (*n* = 8/group; 4 females and 4 males) with a stereotactic frame [48]. Mice in the sham group were injected with phosphate buffered saline (PBS) (*n* = 3, male mice). Tumor growth was monitored using the In Vivo Imaging System (IVIS) and at 14 days post injection, 7/8 mice in both WT and KO groups showed visible tumors and were included in the study. Measuring the tumor size at progressive time points, we found that knockout of AZIN1 resulted in significantly slower tumor growth (Fig. 3C). The survival of the mice was followed, and they were kept alive until they showed clear symptoms of discomfort (Material and Methods). Animals with AZIN1 KO cells had significantly longer survival, with a median survival time ∼20% greater than mice injected with AZIN1 WT cells (Fig. 3D).

Given our prior data demonstrating that AZIN1 overexpression increases cell invasive capacity, we measured tumor dissemination by studying brain sections of mice 14 days after intracranial injection. Tumor identification was facilitated by GFP expression engineered into both the WT and AZIN1 KO D556 cells. To allow for visualization, the sections were stained for DAPI and excited by 488 nm laser to detect GFP expressing cells. As shown in Fig. 3E, at 14 days post injection, the WT D556 cells were observed or detected in other parts of the brain indicating tumor dissemination while KO cell-injected mice did not exhibit tumor dissemination. Observing the inner frontal region of mice skulls (15 days post- injection), we detected features suggestive of mass formation (Fig. 3F). To investigate whether these were the D556 cells that had disseminated to the inner frontal region of the skull, we looked for GFP expressing cells in skull sections and found disseminated D556 cells in the WT injected group but not in the KO group (Fig. 3G). In summary, these in vivo studies revealed that the AZIN1 KO group had reduced tumor progression and prolonged survival, demonstrating that AZIN1 has a role in MB growth and dissemination.

### Evidence of a compensatory mechanism between AZIN1 and c-Myc proteins in MYC amplified MB cell lines

**Fig. 4:**
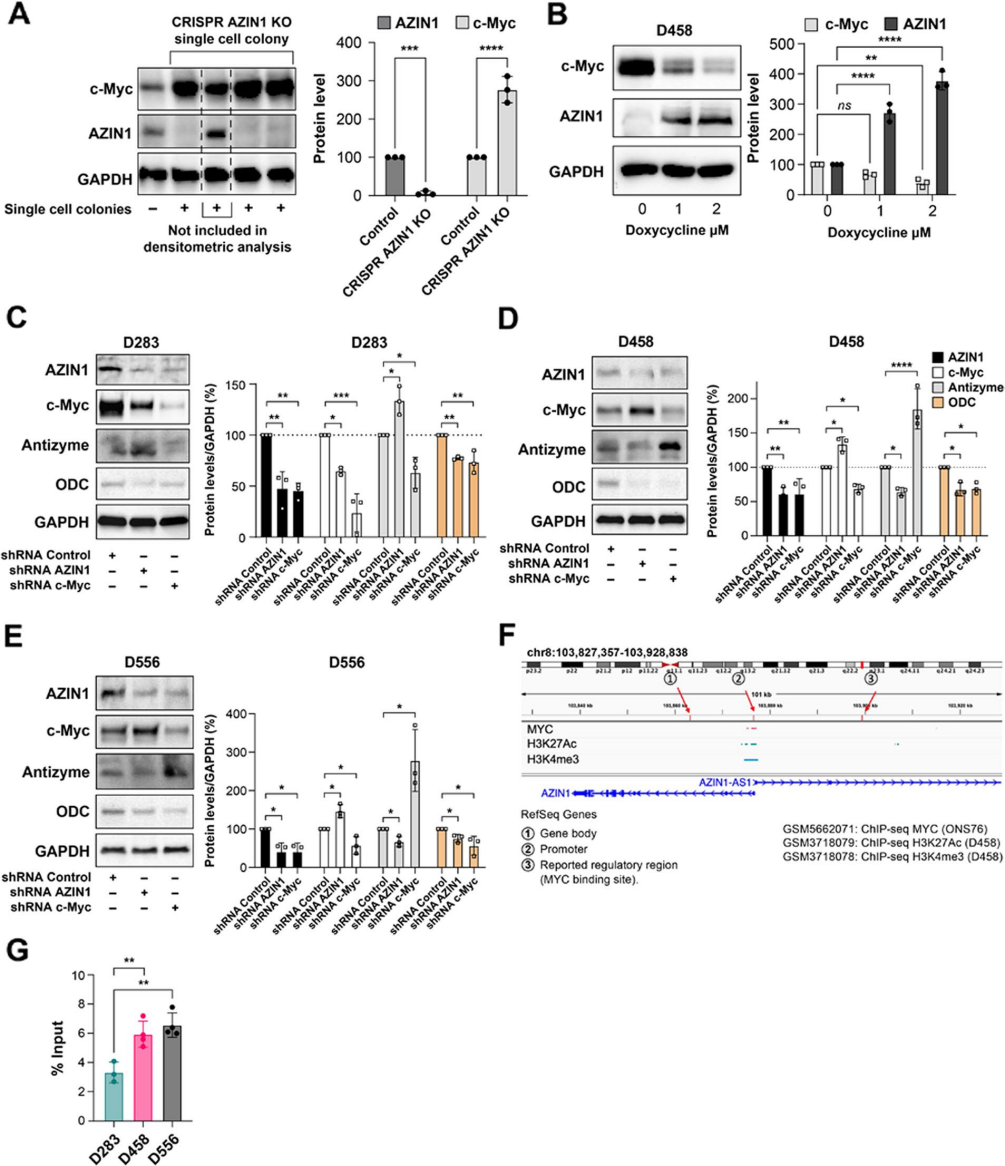

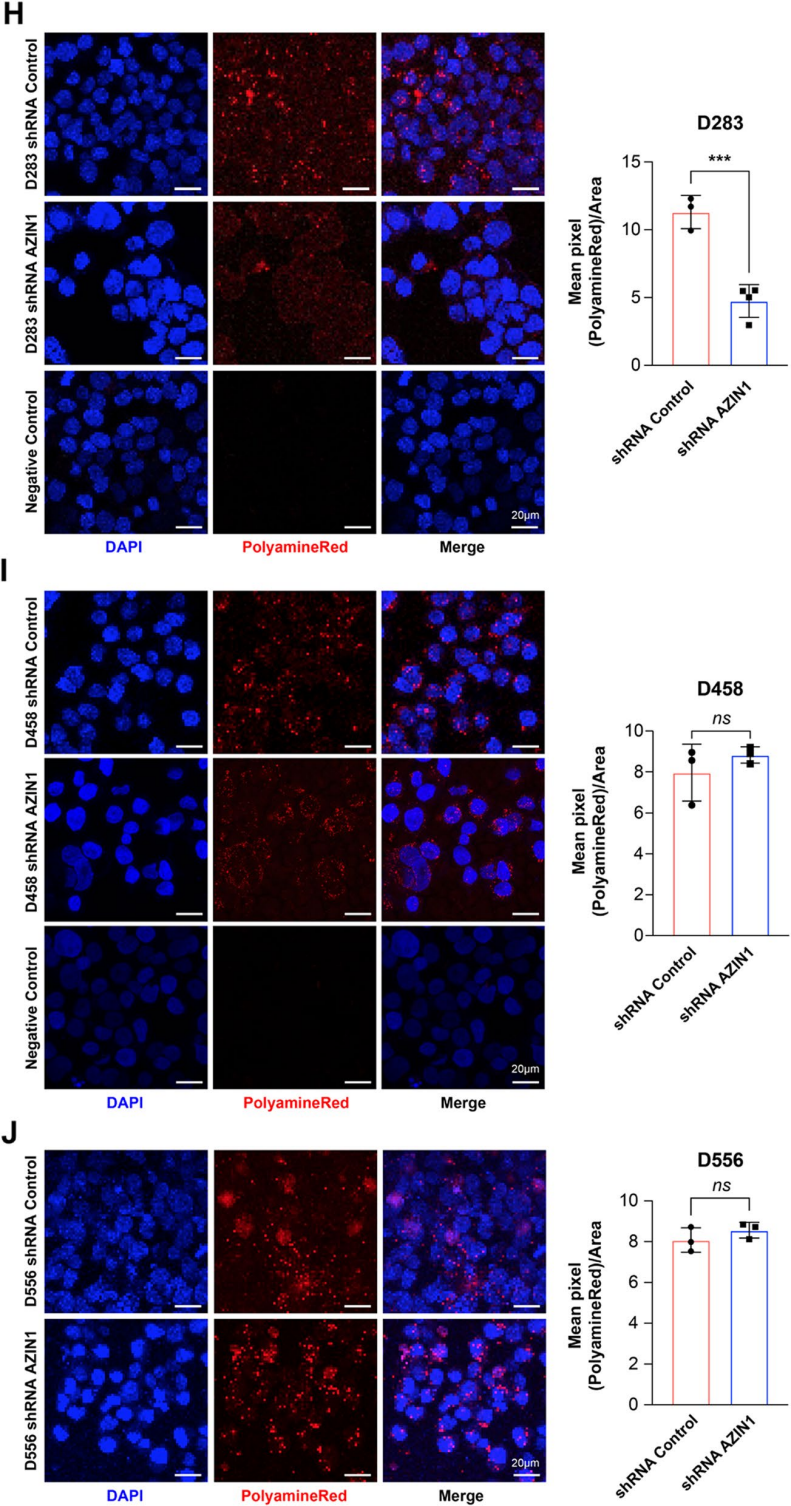
Crosstalk between AZIN1 and c-Myc proteins in MYC amplified cells sustains exogenous polyamine access. **A**-**E** The levels of c-Myc and AZIN1 were measured by WB in MB cell lines for indicated antibodies and GAPDH was used as loading control. **A** Using CRISPR/Cas9 gene editing and specific sgRNAs, AZIN1 protein expression was successfully knocked out in D556 cells. Four single-cell derived colonies are shown, in which three with successful AZIN1 KO. **B** AZIN1 was stably transfected in D458, using an AZIN1 inducible vector. **C** D258, **D** D458 and **E** D556 cells were treated with shRNA Control, shRNA AZIN1 or shRNA c-Myc, after antibiotic selection, cells from passages 2-7 were analyzed. **F **The MYC specific binding motif (5′‐CACGTG‐3′) was identified in the AZIN1 gene; promoter, previously reported MYC binding site and gene body. **G** ChIP assay was performed on the indicated cell lines and the binding between c-Myc and the AZIN1 promotor was analyzed. **H **D283, **I** D458 and **J** D556 were treated with 30 μM of PolyamineRED for 10 min. After incubation, cells were washed three times by PBS, followed by formalin fixation (20 min) and DAPI staining. Using confocal microscopy, images were obtained at Ex/Em=560 nm/585 nm for TAMRA and at Ex/Em=358 nm/461 nm for DAPI. In **A**-**E** and **G**-**J**, bars represent the mean (±S.D.) from three independent experiments. ns stands for no significant difference to indicated controls. * Significant difference to indicated controls (*P* < 0.05), ** (*P*< 0.01), *** (*P*< 0.001), **** (*P*< 0.0001)

We hypothesized that AZIN1 controls c-Myc protein levels through its upstream role on the rate-limiting enzyme of polyamine synthesis, ODC [11] and that, in our model, decreased AZIN1 would result in higher antizyme activity which, in turn, would lead to diminished ODC activity and a subsequent decrease in the c-Myc level. We therefore analyzed the c-Myc protein levels in AZIN1 CRISPR KO D556 cells. Inconsistent with our initial hypothesis, we detected a significant increase in the level of c-Myc (Fig. 4A). We then introduced a doxycycline inducible AZIN1 vector in the D458 cells and found that higher levels of AZIN1 also decrease the c-Myc level (Fig. 4B) indicating crosstalk between AZIN1 and c-Myc proteins in these MYC amplified cell lines. To study this crosstalk in more detail, we created nine different cell lines using shRNA lentiviral constructs. We successfully knocked down AZIN1 or c-Myc in D283, D458, and D556 respectively, and compared them to their own shRNA control lines. As shown in Fig. 4C, in the MYC non-amplified D283 cells, downregulation of AZIN1 leads to reduction of c-Myc levels and c-Myc downregulation led to a decrease of AZIN1 levels. In this cell line, polyamine synthesis, as measured by higher antizyme and lower ODC, can be regulated by targeting AZIN1, leading to a lower c-Myc level. In contrast, in the MYC amplified lines, targeting reductions of AZIN1 leads to increased c-Myc levels (Fig. 4D and E), while c-Myc knock down results in significantly lower AZIN1 levels. Using an alternative mechanism of blocking AZIN1 with shRNA also did not affect polyamine synthesis indicating the existence of a mechanism that could compensate for the lowered AZIN1 in these cell lines through an upregulation of c-Myc. However, shRNA blockade of c-Myc resulted in a clear effect on polyamine synthesis, significantly lowering AZIN1 and ODC while increasing antizyme. In aggregate, these data demonstrate that MYC amplified MB cells exhibit resiliency to AZIN1 blockade through the engagement of an AZIN1 independent/MYC dependent pathway to drive polyamine synthesis.

### c-Myc binds to the promoter region of the AZIN1 gene

To better understand the mechanism underlying the c-Myc knockdown mediated decrease in AZIN1 levels, we hypothesized that c-Myc may act as a transcription factor for the *AZIN1* gene. MYC is a known transcription factor that recognizes and binds to the E-Box motif in DNA [49]. The specific 5′-CACGTG‐3′ sequence has been described as the MYC binding motif, and it is localized primarily in promoters and enhancers of targeting genes [49]. One potential MYC binding site within the *AZIN1* regulatory regions has been previously identified by a genome-wide screen using a specific CRISPR/Cas9 library designed to recognize the CACGTG sequence repeat [50]. This same CACGTG binding motif is present in the *ODC* coding region [51], and the CACGTG repeat is necessary for c-Myc regulation of the *ODC* gene at the transcription initiation stage [10]. *AZIN1* and *ODC* have large sequence similarities [52, 53]. We therefore next checked for CACGTG repeats at *AZIN1* regulatory, promotor, enhancer, and coding regions. We discovered that *AZIN1* has a potential MYC binding site at the promoter region (Fig. 4F and Table 1) and we identified yet additional possible MYC binding sites at the *AZIN1* gene coding region (Fig. 4F and Table 1). In addition, we utilized publicly available ChIP-seq data sets to identify additional MYC binding sites at *AZIN1*. Specifically, we examined MYC binding (ONS76 cell line, GSM5662071), as well as enrichment of active enhancer mark H3K27Ac (D458 cell line, GSM3718079) and active promoter mark H3K4me3 (D458 cell line, GSM3718078) at AZIN1 locus (Fig. 4F). These findings indicated that in MB cells, MYC binds only at the *AZIN1* promoter where it orchestrates transactivation, as evidenced by the enrichment of active histone modification marks.

Next, we designed different primer pairs for each defined site and performed ChIP with the c-Myc antibody. ChIP DNA was then analyzed by qPCR (Table 1). Consistent with the public data, we found that c-Myc binds only to the promotor region of the *AZIN1* gene and notably, that this binding is significantly higher in MYC amplified cell lines (Fig. 4G). Except for the promotor site, c-Myc is not enriched at any other regions of *AZIN1*. Taken together, these data demonstrate that through binding to the *AZIN1* promotor, c-Myc regulates *AZIN1* expression at the transcriptional level, and this upregulation is greater in the MYC amplified cell lines.

### Mechanism underlying increased c-Myc levels in AZIN1 knockdown MB cells with MYC amplification

Polyamines control the expression of several oncogenes, including c-Myc [54]. The amount of polyamines in a cell can change as a result of de novo synthesis (controlled by AZIN1 and ODC), or alternatively, polyamines can be imported from the extracellular space through a polyamine transporter in the cell membrane [54, 55], a secondary process independent of AZIN1 and ODC levels. Given the regulatory role linking polyamines and c-Myc, we analyzed polyamine levels in our cell lines.

We examined the MB cell lines D458 and D556, as they maintain high c-Myc levels even after AZIN1 knock down, and contrasted them with D258, a MB cell line that does not overexpress c-Myc. Using a polyamine specific dye, PolyamineRed, a tetramethylrhodamine-conjugated (TAMRA) derivative of glycine propargyl ester that has cell-penetrating properties and selectively binds to intracellular polyamines and labels them with a red fluorescent dye, we were able to measure polyamine levels [56]. Treating shRNA control and shRNA AZIN1 D283 cells with PolyamineRed, we found that in D258 cells, AZIN1 knock down results in lower intracellular polyamines, indicative of inhibition of polyamine synthesis, with concomitant lowering of c-Myc levels (Fig. 4H). In contrast, blockade of AZIN1 with shRNA failed to lower the intracellular polyamines in D458 and D556 cells (Fig. 4I and J). These cells managed to maintain a high polyamine level, implicating use of the endocytic alternative pathway facilitating the continuously high c-Myc levels despite AZIN1 knock down. In combination, these data reveal that c-Myc regulates AZIN1 at the transcriptional level through binding to its promotor and that MYC-amplified cells are able to maintain high levels of polyamines utilizing a secondary mechanism other than direct synthesis, such as endocytosis.

### AZIN1 is present in the CSF and urine of MB patients suggesting its potential role as a non-invasive biomarker of MB

**Fig. 5:**
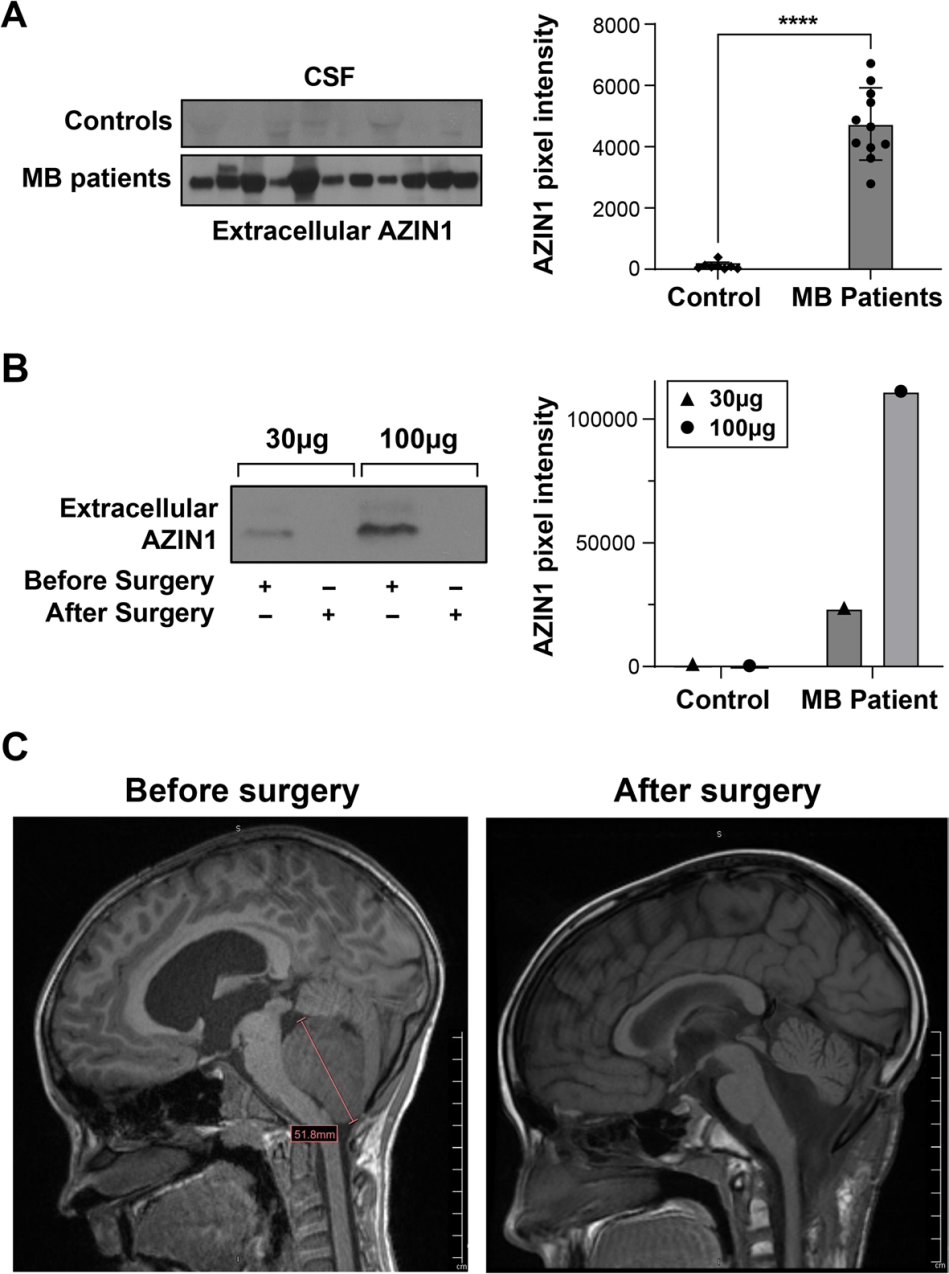
AZIN1 is expressed in the CSF and urine of MB patients. **A** CSF AZIN1 levels were quantified by WB and compared between children with MB (*n* = 11) and age matched fatty filum controls (*n* = 8). **B** Pre- and postoperative urinary AZIN1 levels from one patient, with corresponding MRI **C** at times of urine collection (pre-op and 8 weeks post-op), tumor marked with a red line. Urinary AZIN1 levels were quantified by WB, 30 or 100µg of protein were loaded. In **A**, bars represent the mean (±S.D.) from three independent experiments. Significant difference to indicated controls **** (*P*< 0.0001)

We have previously identified biomarkers that have the potential to detect different types of brain tumors including but not limited to, MB [32, 57]. We tested whether AZIN1 could serve as a biomarker for MB. Analyzing CSF from 11 MB patients (10 with MYC amplifications and 1 unknown MYC status) and comparing them with 8 samples from patients with fatty filum as controls, we found AZIN1 to be significantly higher in the MB patients compared to the controls (Fig. 5A and Supplementary Fig. 4A). Since drawing CSF from patients is considered to be an invasive procedure, as a proof of concept, we quantified AZIN1 in the pre-and post-tumor resection surgery urine samples of two MB patients (with MYC amplification status). Urinary AZIN1 levels were increased pre-surgery and then significantly decreased at 8 weeks post-tumor resection surgery, (data from one patient is presented in Fig. 5B and C, and an additional patient is added and presented in Supplementary Fig. 4B). These initial and limited data suggest that AZIN1 may represent a novel biomarker for MB patient diagnosis and clinical follow-up. Furthermore, these results confirm that in vivo, AZIN1 is secreted from the MB tissue, corroborating our in vitro results presented in Fig. 2.

## Discussion

Despite recent advances, MB remains a significant cause of morbidity and mortality for children and adults, particularly with regard to refractory subgroups of MB that remain with unacceptably high fatality rates. Consequently, there is a profound clinical need to better understand the mechanisms that drive MB aggressiveness and to identify new targets that may provide novel treatments. Concordant with these goals, we have identified the molecule AZIN1, which promotes tumorigenesis in other cancers, (among them hepatocellular carcinoma [58], colon [59], lung [60] and prostate cancer [31]), to be highly expressed in MB tissues and have established that AZIN1 induces increased proliferation, invasion, and colony formation in MB cells. These results implicate AZIN1 to be involved in MB pathogenesis and support the idea that a strategy that targets AZIN1-mediated effects might provide an innovative approach to the treatment of MB.

Importantly, our research revealed that AZIN1 expression is significantly correlated with MYC amplification. The AZIN1 levels are also higher in MYC-amplified cell lines. In contrast, the D283 MYC non-amplified cell line harbors five copies of the AZIN1 gene, yet it expresses a low levels of AZIN1 and c-Myc. This line exhibits significantly lower c-Myc enrichment at the AZIN1 promoter. This diminished c-Myc binding likely contributes to the reduced transcriptional activity of AZIN1, ultimately leading to lower protein expression compared to MYC-amplified cell lines. This finding highlights the importance of the c-Myc transcriptional activity for AZIN1 protein expression.

MYC amplification is one important molecular characteristics of the most aggressive and refractory MB subtypes [16] and is associated with metastasis [17], treatment failure and poor prognosis [19]. Given the significant challenges in directly targeting MYC, AZIN1 targeting may provide a novel, alternative approach to favorably modulating the aggressiveness induced by this key oncogene [11, 61]. c-Myc function requires active ODC and polyamine-mediated macromolecule stabilization which are regulated by AZIN1 [11, 13, 14, 18] and our in vivo data support the potential utility of this approach. Blockade of AZIN1 resulted in a significant delay of tumor progression and prolonged survival, even when using a MYC amplified MB tumor line.

**Fig. 6:**
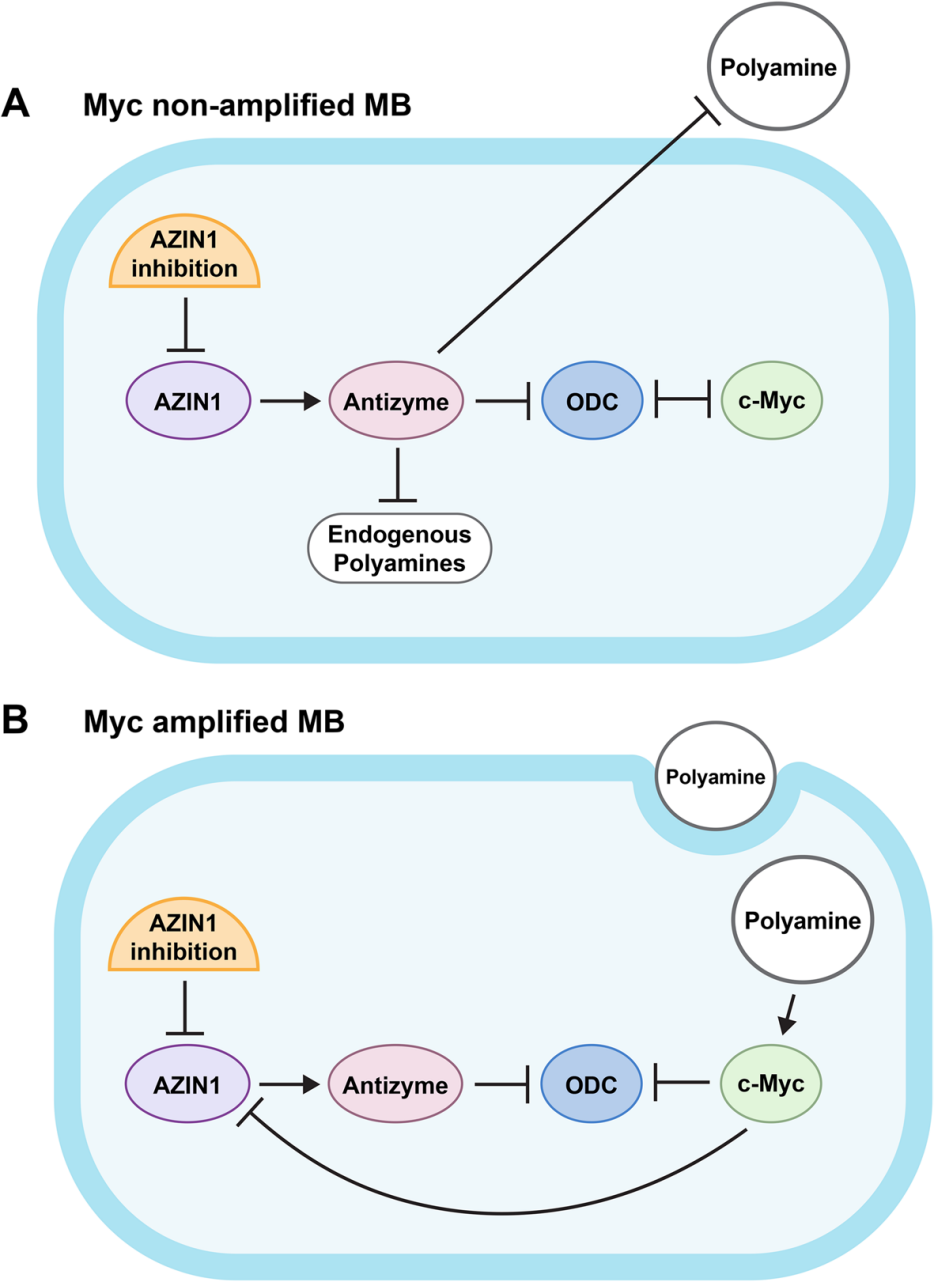
The role of AZIN1 in the regulation of the MB phenotype. **A**, in MYC non amplified MB, targeting AZIN1 leads to inhibition of the tumor suppressor Antizyme facilitates the degradation of several growth promoters, including ODC and also blocks the polyamine endocytoses. AZIN1 also regulates c-Myc through ODC. **B**, in MYC amplified MB, targeting AZIN1 leads to increased c-Myc levels. In a positive-feedback loop, c-Myc amplification sustains active polyamine endocytoses, leading to an increase c-Myc gene expression. In **A** and **B**, as c-Myc binds to the promotor region of AZIN1, inhibiting c-Myc leads to decrease of AZIN1

Prior work has explored targeting links in the AZIN1 pathway such as ODC. Specific ODC inhibitors such as D, L-alpha-difluoromethylornithine (DFMO) [61, 62] have seen modest anti-tumor efficacy in the clinic [11, 63–65]. A potential advantage of targeting AZIN1 for MB therapy [66, 67] is that AZIN1 has a broader impact on the metabolic pathway that drives tumorigenesis, encompassing ODC along with other related molecules [68]. Supporting this premise, our data shows that c-Myc levels remain high in AZIN1 knockdown MYC amplified MB cells, yet targeting AZIN1 resulted in lowered cell proliferation, highlighting the centrality of the growth promoting role of AZIN1 (Fig. 6).

As part of our investigation into the role of AZIN1 in MB tumorigenesis, we identified a regulatory circuit between AZIN1 and c-Myc, in which c-Myc governs AZIN1 expression by binding to its promotor region. In parallel, AZIN1 also regulates c-Myc through control of polyamine synthesis in MYC non amplified cells, but this system appears to be dysregulated in cells with MYC amplification. In cells with MYC amplification, c-Myc bypasses AZIN1-controlled polyamine synthesis and maintains access to high level of polyamines, likely through the initiation of an alternative endocytosis mechanism that imports exogenous polyamines. Consequently, these data suggest that a dual inhibition strategy in which both AZIN1 and polyamine endocytosis are targeted simultaneously might be a novel approach to be considered in MYC amplified tumor subgroups (Fig. 6).

Also relevant to better understanding the pathogenesis of MB, to our knowledge, we are the first to demonstrate that AZIN1 is a protein secreted via EVs. Furthermore, we found that MB-derived EVs carried AZIN1 as a membrane protein, can transfer it to target MB cells, and can significantly increase their invasion in vitro. This is also the first time that AZIN1 has been demonstrated to be part of the structure of MB-derived EVs, and to be secreted by them and to play a role in MB cell invasion. While the role of AZIN1-EVs needs to be further elucidated, it could be speculated that it may promote MB local invasion and metastasis, which in MB typically occurs in other areas of the CNS via the CSF. This hypothesis is further supported by our results demonstrating that, compared to controls, CSF samples from MB patients are characterized by significantly higher levels of AZIN1. Importantly, AZIN1 was also detectable, and significantly higher, in the urine sample of MB patients prior to resection surgery compared to the follow-up sample collected after tumor resection surgery, corroborating our hypothesis that secreted AZIN1 is mainly sourced from the primary MB lesion. While both CSF and urinary AZIN1 data require validation using larger sample sizes representative of all four different MB subgroups and from longitudinal studies, these preliminary findings suggest that AZIN1 may have a role as a diagnostic, prognostic, and clinical follow-up biomarker for MB.

While AZIN1 has been associated with several tumors, there are no standalone studies of AZIN1 in brain tumor pathogenesis - an important distinction of the work presented here, given the outsized impact of brain tumors on pediatric life expectancy. Moreover, while AZIN1 regulation of polyamine synthesis is a general mechanism that can be implicated in many pathways relevant to tumorigenesis (and recognizing that other mechanisms, such as RNA modification, may also contribute to AZIN1-related tumor development), our discovery of a direct, specific c-Myc/AZIN1 negative feedback loop is a novel finding that provides an mechanistic insight with potential therapeutic implications [69]. To our knowledge, this is the first time that AZIN1 has been associated with MB. Data from our work show that AZIN1 is highly expressed in group 3 MB, induces cellular aggressiveness, and is associated with MYC amplified status. AZIN1 is secreted in patients with MB and its measured levels of urinary and CSF AZIN1 correlate with tumor status. These findings implicate AZIN1 in MB pathogenesis and suggest that further study of this molecule as a potential therapeutic target may be warranted.

## Supplementary Information

Supplementary Material 1: Supplemental Figure 1. Significant correlations between c-Myc mRNA and AZIN1 downstream genes and amplification status. Using the publicly available dataset of 223 MB patients [43] and analyzing the correlation between; A ODC1 vs c-Myc mRNA expressions (*R = 0.239*, *p = 3.15 e-04*) and B OAZ1 vs c-Myc mRNA (*R = 0.331*, *p = 4.15 e-07*). Analyzing the correlation between AZIN1 and MYC mRNA in publicly available datasets of; C 763 patients [40] (*R=0.171*, *p = 2.08e-06*), D 167 patients [8, 41, 43] (*R = 0.167*, *p = 0.033*) and E 420 patients [42] (*R = 0.199*, *p = 4.09 e-05*). F Using the publicly available CCLE dataset of 1037 cell lines and analyzing the correlation between AZIN1 and MYC mRNA (*R = 0.086*, *R2 = 0.007,* *p = 0.0057*). Using the CCLE database, the correlation between MYC and AZIN1 copy numbers in; G MYC amplified cell lines (*n*= 638) (*R = 0.49*, *R*^2^ *= 0.24,* *p = 4.861007e-40*), and H AZIN1 amplified cell lines (*n*= 559) (*R = 0.68*, *R*^2^ *= 0.47,* *p = 9.094104e-78*) were analyzed.

Supplementary Material 2: Supplementary Figure 2. AZIN1 is detected in the cell media of MB cell lines. Normal human fetal glial SVG, D283, D458 and D556 cells were cultured for 24h and serum starved for additional 24h, extracellular AZIN1 was detected by ELISA.

Supplementary Material 3: Supplementary Figure 3. Confirmation of AZIN1 knockout in D556 cell line. A Selected gRNAs and their locations within the exon 3 of AZIN1 gene. B Gel image of PCR shows the knockout efficiency of pooled cells transfected with gRNA1+2 and gRNA1+3. C Sanger sequencing of homozygous AZIN1 KO clones in D556-Luc-GFP cells, showing four single cells clones derived lines (C6, E4, E5 and F8) with frame shifts. D Gel image of PCR screening of single clones shows clones E4, and C6 are homozygous AZIN1 KO candidate clones. (E) MTT assay of D556 AZIN1 KO cells treated with AZIN1-EVs and AZIN1KO-EVs for 24, 48, 72 and 96 hours.

Supplementary Material 4: Supplementary Figure 4. AZIN1 is expressed in CSF and urine of MB patients. A CSF AZIN1 levels were quantified ELISA and compared between children with MB (*n* = 11) and age matched fatty filum controls (*n* = 8). B Pre- and postoperative urinary AZIN1 levels from two patients (with MYC amplification status). The urine collection was performed pre-operation and 8 weeks post-operation patient 1 (and 11 weeks patient 2). Urinary AZIN1 levels were quantified by ELISA. In A, bars represent the mean (±S.D.) from three independent experiments. * Significant difference to indicated controls

Supplementary Material 5: Supplementary Table 1. Single-Cell RNA sequencing analysis for AZIN1 expression. In A and B, Analyzing MB subgroups individually, only in group 3 MB the AZIN1 expression is significantly higher MYC positive cells verses MYC negative. Mean expression, lower CI, Upper CI and adjusted *p* values of the data of Figure 1M is shown. In C, Comparing AZIN1 expression in MYC positive cells between the MB subgroups. The MYC positive cells were subset by cells where MYC expression > 0 (i.e. log-normalized(MYC-expression) > 0). Pairwise statistical comparisons between groups were performed using the Student's t-test. Additionally a *one-way ANOVA-Kruskal-Wallis test was applied to evaluate significant differences in AZIN1 expression across all groups. Since the *ANOVA-test *p*.value were 2.36e-07, we performed **Post-hoc Test (Tukey's HSD) for pairwise comparison between subgroups to identify differences (C).

Supplementary Material 6: Supplementary Video 1. AZIN1 is released through EVs in D283 cells. pCDNA3.1-Clover-AZIN1 plasmid was transfected in D283 cells and analyzed by confocal microscopy, Multiple images are taken in Z-stack and 3D images are shown in video format.

Supplementary Material 7: Supplementary Video 2. AZIN1 is released through EVs in D458 cells. pCDNA3.1-Clover-AZIN1 plasmid was transfected in D458 cells and analyzed by confocal microscopy, Multiple images are taken in Z-stack and 3D images are shown in video format.

Supplementary Material 8: Supplementary Video 3. AZIN1 is released through EVs in D556 cells. pCDNA3.1-Clover-AZIN1 plasmid was transfected in D556 cells and analyzed by confocal microscopy, Multiple images are taken in Z-stack and 3D images are shown in video format.

## Data Availability

No datasets were generated or analysed during the current study.
