# Supplementary figures and images for: AZIN1 level is increased in medulloblastoma and correlates with c-Myc activity and tumor phenotype
Source: J Exp Clin Cancer Res. 2025 Feb 17;44:56. doi: 10.1186/s13046-025-03274-1 (PMC11831846; doi:10.1186/s13046-025-03274-1)

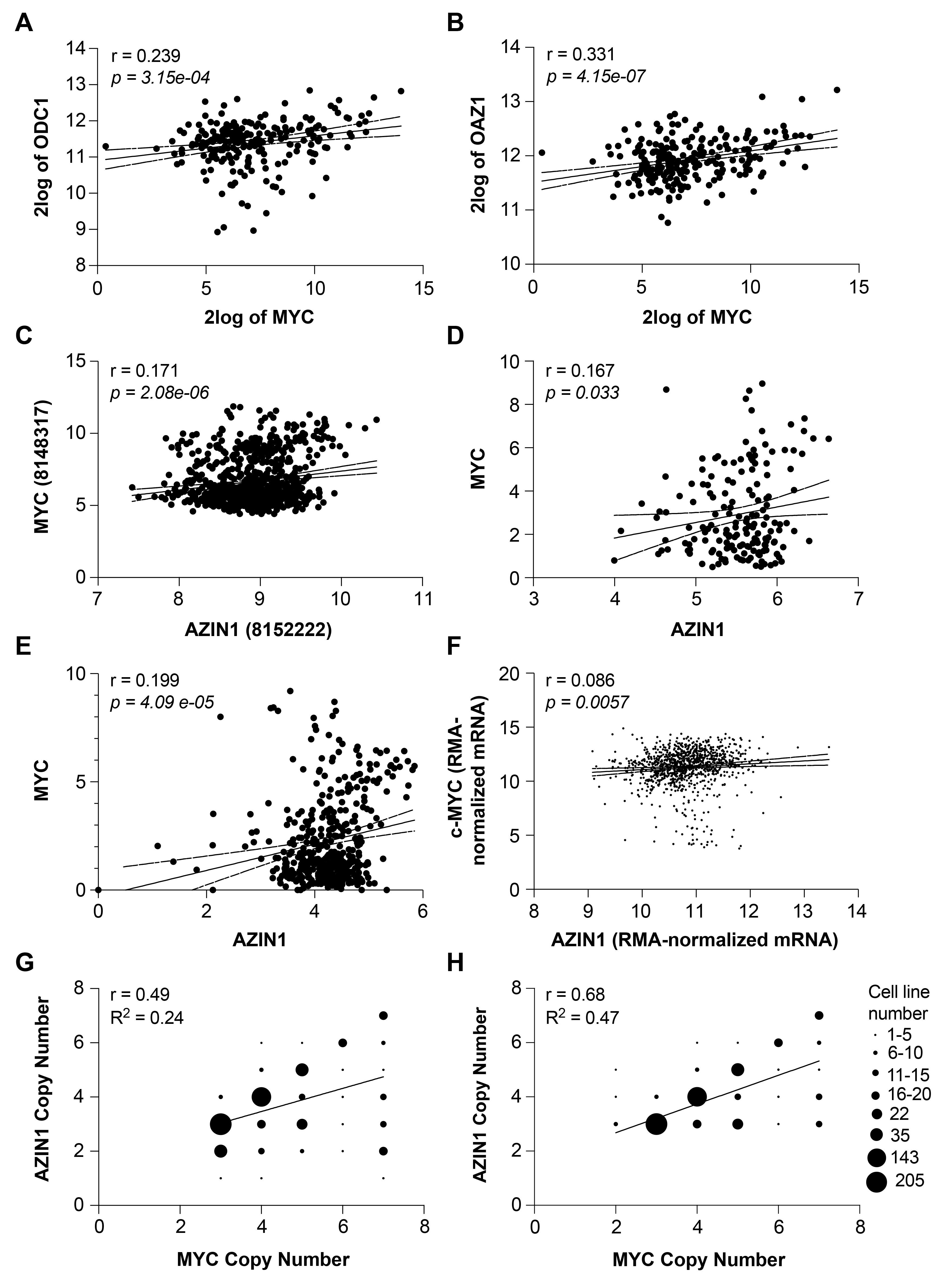

Supplement: Supplementary file 1 — Supplementary Material 1: Supplemental Figure 1. Significant correlations between c-Myc mRNA and AZIN1 downstream genes and amplification status. Using the publicly available dataset of 223 MB patients [43] and analyzing the correlation between; A ODC1 vs c-Myc mRNA expressions (R = 0.239, p = 3.15 e-04) and B OAZ1 vs c-Myc mRNA (R = 0.331, p = 4.15 e-07). Analyzing the correlation between AZIN1 and MYC mRNA in publicly available datasets of; C 763 patients [40] (R=0.171, p = 2.08e-06), D 167 patients [8, 41, 43] (R = 0.167, p = 0.033) and E 420 patients [42] (R = 0.199, p = 4.09 e-05). F Using the publicly available CCLE dataset of 1037 cell lines and analyzing the correlation between AZIN1 and MYC mRNA (R = 0.086, R2 = 0.007, p = 0.0057). Using the CCLE database, the correlation between MYC and AZIN1 copy numbers in; G MYC amplified cell lines (n= 638) (R = 0.49, R2 = 0.24, p = 4.861007e-40), and H AZIN1 amplified cell lines (n= 559) (R = 0.68, R2 = 0.47, p = 9.094104e-78) were analyzed. [file 13046_2025_3274_MOESM1_ESM.tif]

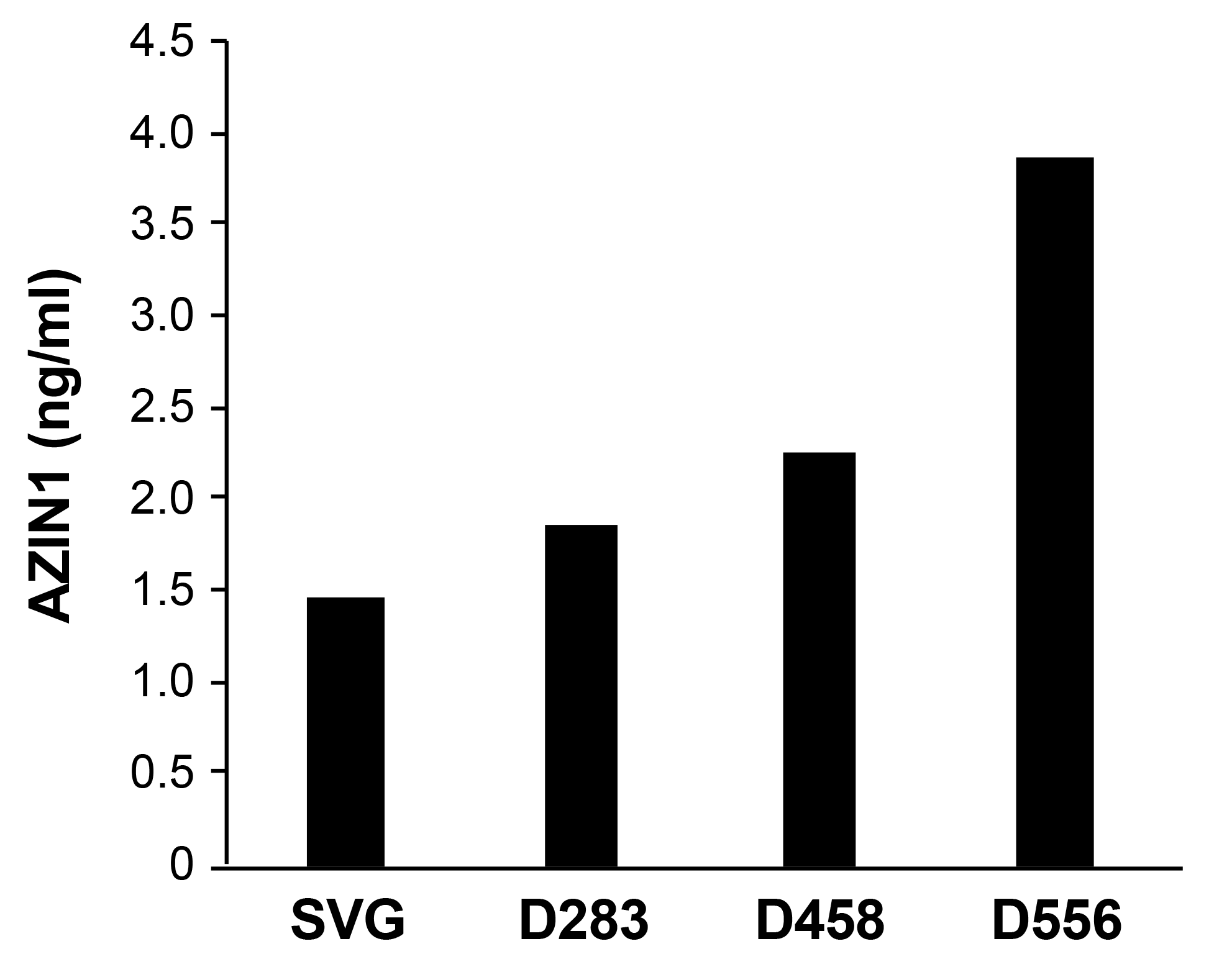

Supplement: Supplementary file 2 — Supplementary Material 2: Supplementary Figure 2. AZIN1 is detected in the cell media of MB cell lines. Normal human fetal glial SVG, D283, D458 and D556 cells were cultured for 24h and serum starved for additional 24h, extracellular AZIN1 was detected by ELISA. [file 13046_2025_3274_MOESM2_ESM.tif]

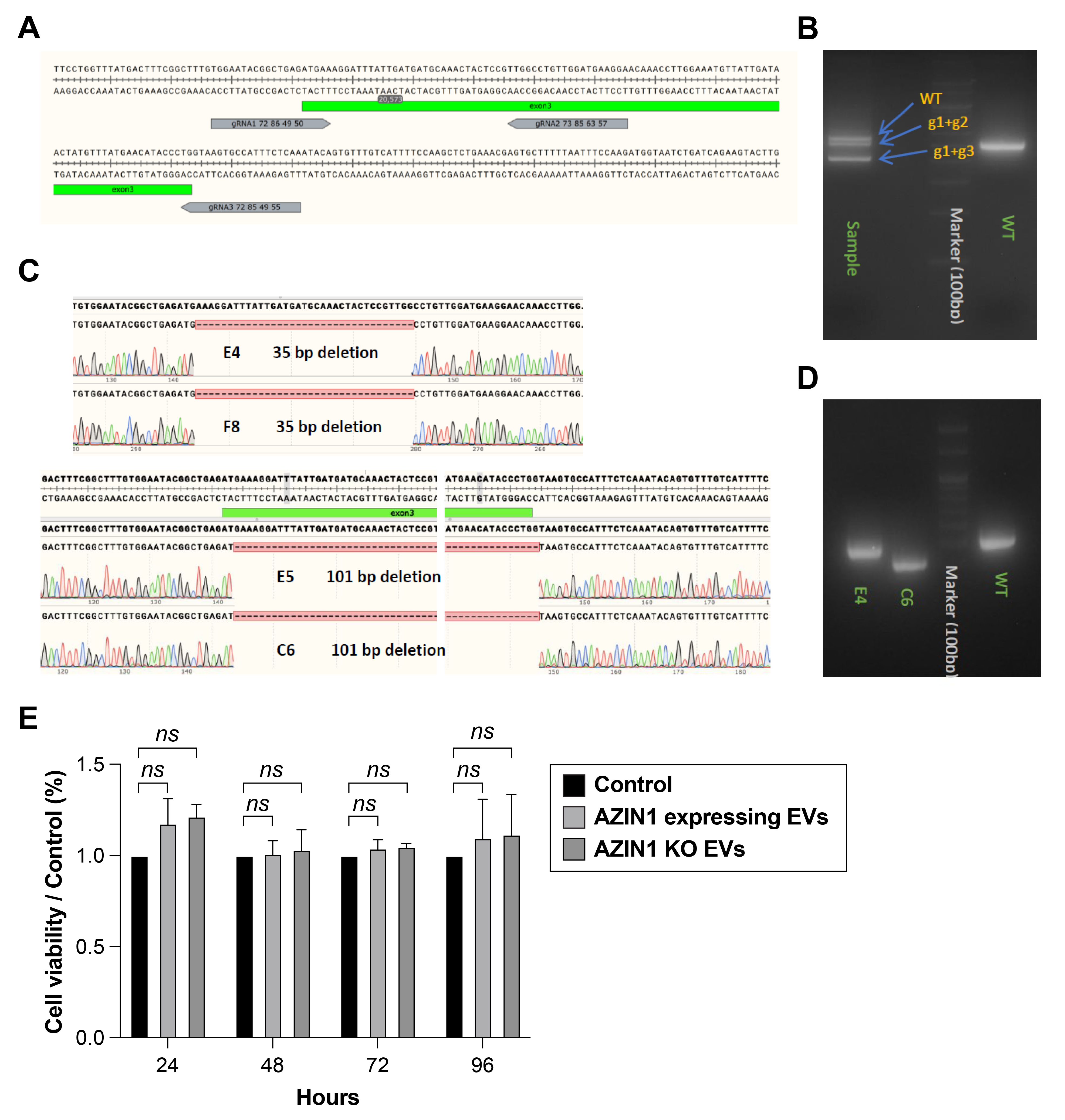

Supplement: Supplementary file 3 — Supplementary Material 3: Supplementary Figure 3. Confirmation of AZIN1 knockout in D556 cell line. A Selected gRNAs and their locations within the exon 3 of AZIN1 gene. B Gel image of PCR shows the knockout efficiency of pooled cells transfected with gRNA1+2 and gRNA1+3. C Sanger sequencing of homozygous AZIN1 KO clones in D556-Luc-GFP cells, showing four single cells clones derived lines (C6, E4, E5 and F8) with frame shifts. D Gel image of PCR screening of single clones shows clones E4, and C6 are homozygous AZIN1 KO candidate clones. (E) MTT assay of D556 AZIN1 KO cells treated with AZIN1-EVs and AZIN1KO-EVs for 24, 48, 72 and 96 hours. [file 13046_2025_3274_MOESM3_ESM.tif]

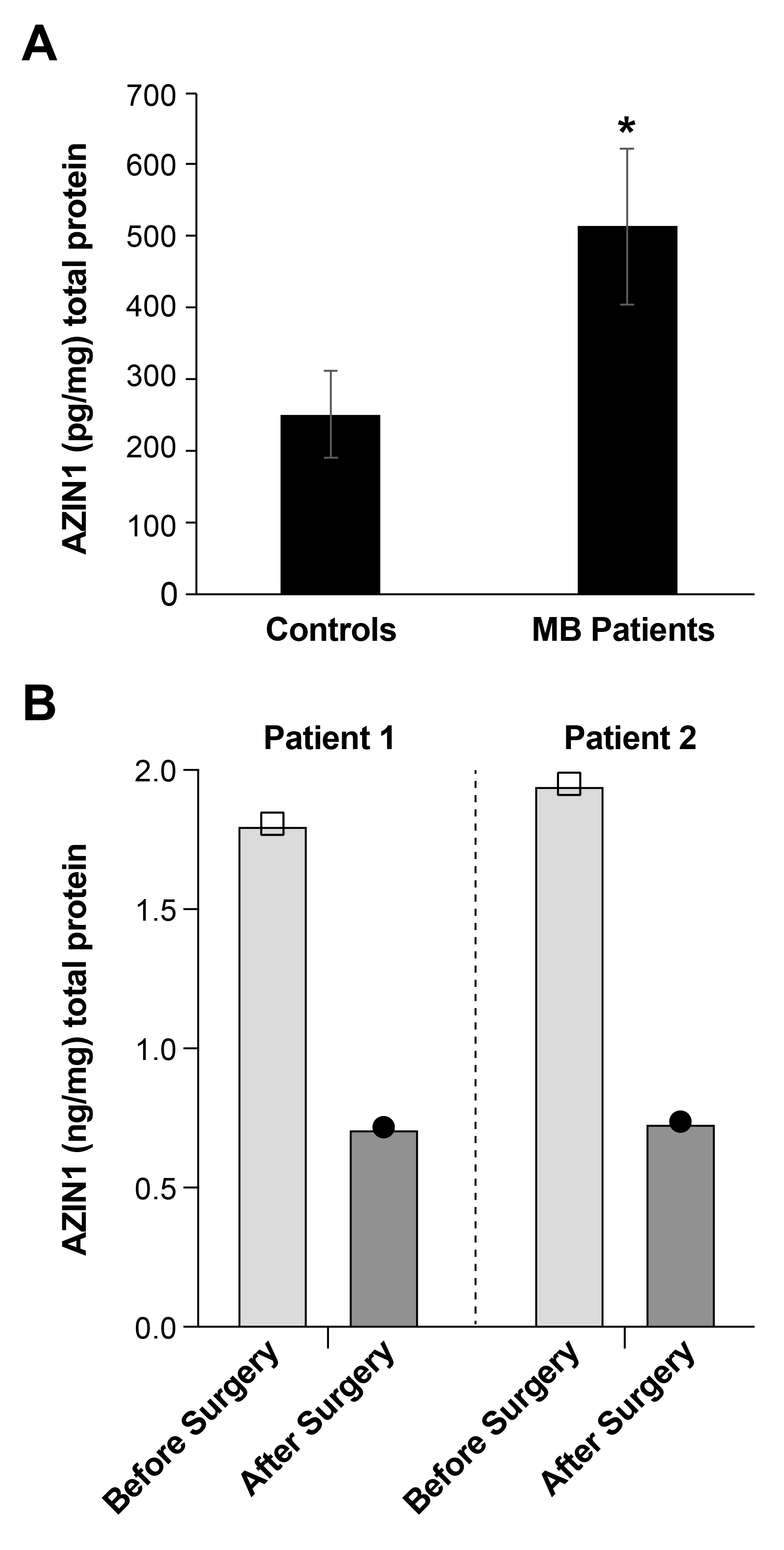

Supplement: Supplementary file 4 — Supplementary Material 4: Supplementary Figure 4. AZIN1 is expressed in CSF and urine of MB patients. A CSF AZIN1 levels were quantified ELISA and compared between children with MB (n = 11) and age matched fatty filum controls (n = 8). B Pre- and postoperative urinary AZIN1 levels from two patients (with MYC amplification status). The urine collection was performed pre-operation and 8 weeks post-operation patient 1 (and 11 weeks patient 2). Urinary AZIN1 levels were quantified by ELISA. In A, bars represent the mean (±S.D.) from three independent experiments. * Significant difference to indicated controls [file 13046_2025_3274_MOESM4_ESM.tif]
