# Supplementary material for: AZIN1 level is increased in medulloblastoma and correlates with c-Myc activity and tumor phenotype
Source: J Exp Clin Cancer Res. 2025 Feb 17;44:56. doi: 10.1186/s13046-025-03274-1 (PMC11831846; doi:10.1186/s13046-025-03274-1)
Supplement: Supplementary file 5 — Supplementary Material 5: Supplementary Table 1. Single-Cell RNA sequencing analysis for AZIN1 expression. In A and B, Analyzing MB subgroups individually, only in group 3 MB the AZIN1 expression is significantly higher MYC positive cells verses MYC negative. Mean expression, lower CI, Upper CI and adjusted p values of the data of Figure 1M is shown. In C, Comparing AZIN1 expression in MYC positive cells between the MB subgroups. The MYC positive cells were subset by cells where MYC expression > 0 (i.e. log-normalized(MYC-expression) > 0). Pairwise statistical comparisons between groups were performed using the Student's t-test. Additionally a *one-way ANOVA-Kruskal-Wallis test was applied to evaluate significant differences in AZIN1 expression across all groups. Since the *ANOVA-test p.value were 2.36e-07, we performed **Post-hoc Test (Tukey's HSD) for pairwise comparison between subgroups to identify differences (C). [file 13046_2025_3274_MOESM5_ESM.docx]

| **group** | **MYC status** | **p.adj** |
| --- | --- | --- |
| GR3 | MYC Positive vs. MYC Negative | 0.000023 |
| GR4 | MYC Positive vs. MYC Negative | 0.37 |
| SHH | MYC Positive vs. MYC Negative | 0.8 |
| WNT | MYC Positive vs. MYC Negative | 0.4 |


| **Subgroup** | **MYC status** | **AZIN1 expression mean** | **Lower CI** | **Upper CI** |
| --- | --- | --- | --- | --- |
| GR3 | MYC Positive | 0.173 | 0.131 | 0.214 |
| GR3 | MYC Negative | 0.00812 | -0.0558 | 0.072 |
| GR4 | MYC Positive | -0.0242 | -0.147 | 0.0988 |
| GR4 | MYC Negative | -0.0853 | -0.138 | -0.0331 |
| SHH | MYC Positive | -0.127 | -0.319 | 0.0654 |
| SHH | MYC Negative | -0.153 | -0.221 | -0.0848 |
| WNT | MYC Positive | 0.0196 | -0.0299 | 0.0692 |
| WNT | MYC Negative | -0.0188 | -0.0939 | 0.0564 |

| **Group** | **Mean of the raw data** | **Lower CI** | **Upper CI** |
| --- | --- | --- | --- |
| GR3 | 0.173 | 0.131 | 0.214 |
| GR4 | -0.0242 | -0.147 | 0.0988 |
| SHH | -0.127 | -0.319 | 0.0654 |
| WNT | 0.0196 | -0.0299 | 0.0692 |

| **Group** | **Mean difference** | **Lower CI** | **Upper Cl** | **p-adj** |
| --- | --- | --- | --- | --- |
| GR4-GR3 | -0.1968971 | -0.3444 | -0.0494151 | 0.0034006 |
| SHH-GR3 | -0.2997665 | -0.5125 | -0.0870137 | 0.0016905 |
| WNT-GR3 | -0.1531106 | -0.2411 | -0.0650864 | 0.0000476 |
| SHH-GR4 | -0.1028694 | -0.3498 | 0.14401857 | 0.7072905 |
| WNT-GR4 | 0.0437865 | -0.1093 | 0.19688177 | 0.8829559 |
| WNT-SHH | 0.1466559 | -0.07 | 0.36333772 | 0.3032333 |
